# The Use of Electrospray Mass Spectrometry to Determine Speciation in a Dynamic Combinatorial Library for Anion Recognition

**DOI:** 10.1002/chem.201201302

**Published:** 2012-09-20

**Authors:** Hazel I A Phillips, Aleksey V Chernikov, Nicholas C Fletcher, Alison E Ashcroft, James R Ault, Maria H Filby, Andrew J Wilson

**Affiliations:** a School of Chemistry and Chemical Engineering, Queen's University BelfastStranmillis Road, Belfast BT9 5AG (UK); b Astbury Centre for Structural Molecular Biology, University of Leeds InstitutionWoodhouse Lane, Leeds LS2 9JT (UK); c School of Chemistry, University of Leeds InstitutionWoodhouse Lane, Leeds LS2 9JT (UK)

**Keywords:** 2,2′-bipyridine, anion recognition, combinatorial chemistry, iron, mass spectrometry

## Abstract

The composition of a dynamic mixture of similar 2,2′-bipyridine complexes of iron(II) bearing either an amide (5-benzylamido-2,2′-bipyridine and 5-(2-methoxyethane)amido-2,2′-bipyridine) or an ester (2,2′-bipyridine-5-carboxylic acid benzylester and 2,2′-bipyridine-5-carboxylic acid 2-methoxyethane ester) side chain have been evaluated by electrospray mass spectroscopy in acetonitrile. The time taken for the complexes to come to equilibrium appears to be dependent on the counteranion, with chloride causing a rapid redistribution of two preformed heteroleptic complexes (of the order of 1 hour), whereas the time it takes in the presence of tetrafluoroborate salts is in excess of 24 h. Similarly the final distribution of products is dependent on the anion present, with the presence of chloride, and to a lesser extent bromide, preferring three amide-functionalized ligands, and a slight preference for an appended benzyl over a methoxyethyl group. Furthermore, for the first time, this study shows that the distribution of a dynamic library of metal complexes monitored by ESI-MS can adapt following the introduction of a different anion, in this case tetrabutylammonium chloride to give the most favoured heteroleptic complex despite the increasing ionic strength of the solution.

## Introduction

Over the last 40 years, the polypyridine complexes of ruthenium(II) have attracted considerable interest as recognition units for a vast array of small molecules,^1^ the structural recognition of DNA,^2^ and more recently, proteins.^3^, ^4^ Their evaluation in the specific detection of anions has spawned a wealth of new complexes with varying degrees of selectivity.^1^, ^5^ Importantly, Uppadine et al. have highlighted that a *C*_3_-symmetric cavity, formed by arranging three 5,5′-functionalized-2,2′-bipyridine ligands around a ruthenium(II) metal centre, could be used to selectively recognize a range of small inorganic anions by hydrogen bonding to the amide groups,^6^ whereas more recently, Wu and Janiak have illustrated that a ruthenium(II) complex, with two carbamate groups on each 2,2′-bipyridine ligand has a good selectivity for sulfate.^7^ Similarly, by using asymmetric ligands, we have explored the influence of the relative position of the three amide groups in two comparable *mer*- and *fac* isomers and the resulting effect on anion recognition.^8^

The synthesis of compounds for anion specific recognition is not trivial. In the case of complexes based on late transition metals, such as ruthenium(II), it can also be prohibitively expensive, both in the cost of the materials and the labour required, making it almost impossible to screen a large number of similar complexes to optimize for selectivity. To overcome this, there has been interest in the use of dynamic combinatorial chemistry (DCC).^9^–^13^ This creates a library of compounds that can readily adjust to the optimal conditions within a controlled environment. Such “target induced adaptation and selection”^13^ for the optimal complementarity between a host and a guest has been demonstrated in a variety of systems, with recent examples including species that template around simple cations such as lithium,^14^ calcium^15^ and barium,^16^ polymer-bound ammonium salts^17^ and even carbon dioxide.^18^ The identification of suitable candidates to selectively recognize anionic species such as dihydrogen phosphate^19^ and sulfate^20^, ^21^ have also been reported.

DCC is reliant upon a readily reversible reaction that can adjust the composition of the library by the introduction of an external stimulus.^11^ Labile divalent cations, such as those from the first transition series, have been widely considered in this respect, notably with chelating di- and triimine ligand systems. The pioneering work of Lehn^22^ demonstrated that the structural composition within a library of cyclic helicates^23^ and grids^24^ can be strongly influenced by the presence of certain anions, and the metal ions used. Further, the recent work of Barboiu and co-workers has extended these ideas to self-optimization in networks and lattices.^10^, ^25^ In a similar vein, Constable and co-workers have shown that a *fac* isomer of a cobalt(II) tris-chelate can be amplified over the statistically favoured *mer* form by the reversible interaction with a triamine,^26^ and Sasaki et al. have shown that the concentration of a Λ-*mer* iron(II) complex with appended galactose groups is amplified by the addition of lectin.^27^ Furthermore, a library of copper(II) salicylimides has been shown to adjust concentration in the presence of a RNA olignucleotide.^28^ Anions have also been shown to direct the observed distribution within a family of metal complexes composed of a mixture of ligands, with examples including both terpyridine^29^ and bipyridine^30^, ^31^ systems.

However, the issue of identifying the relative abundance of the various components within a dynamic combinatorial library (DCL) remains problematic.^11^ In the case of metal ion-based systems, a variety of techniques have been employed, ranging from reverse-phase HPLC,^28^ size exclusion chromatography,^32^ CD spectroscopy,^27^ extraction studies,^33^ and ^1^H NMR spectroscopy,^30^ although in the case of iron(II) complexes this is often problematic,^31^ and for cobalt(II) the use of paramagnetic NMR spectroscopy is required.^26^, ^34^ Even then, in the case of ion paired species, the spectroscopic studies can be misleading.^35^ To overcome this, interrogation of dynamic systems has been attempted using electrospray mass spectrometry (ESI-MS).^12^, ^14^, ^22^–^24^, ^32^, ^36^ In general the speciation of mixtures of metal complexes by mass spectrometry has been shown to provide reliable data,^37^ even in dynamic systems.^38^ However, it is not without significant drawbacks, such as the necessity to assume that the relative composition of the solution and the gas phases are comparable. The ionization process, which by necessity, separates an ion pair and strips off the solvation sphere, could potentially give a misleading result. It is also assumed that each of the species analysed give a similar detectable response in the instrument. As a result the data can at best only be considered qualitative without making a number of assumptions and careful consideration of the ligand systems involved.^37^ Nevertheless, it has been shown to provide some very informative data. For example, Schröder and co-workers have recently evaluated the composition of a DMF solution of the late 3d metal ions^39^ and significantly demonstrated size selective anion binding by using ESI-MS.^40^

In constructing an adaptive DCL for the recognition of anions, it is worth considering structures in which the anion has already been shown to provide a template for the structure. In particular, examples of *C*_3_-symmetric structures with labile metal centres including zinc(II), iron(II) and cobalt(II) and diimine ligands providing suitable cavities to enclose small inorganic anions are prevalent in the literature.^7^, ^21^, ^31^, ^41^ Of these, iron(II) complexes of 2,2′-bipyridine have previously been shown to be appropriate to dynamic studies,^27^ with complexes having been shown to selectivity adapt and recognize heparin.^42^ Similarly, the electrospray detection of a variety of iron(II) bipyridine complexes has been reported^43^ providing a robust platform from which we can construct a DCL to investigate whether it is possible to discern an appropriate assembly to selectively recognize a specific anion.

In the following account, we wish to validate the use of a DCL by using an iron(II) bipyridine system, monitored by routine ESI-MS, and then, for the first time, use it to instruct the observer of the appropriate ligand combination for anion binding. This can be then compared with our previously reported findings using inert ruthenium(II) complexes, but without the necessity to separate, isolate or synthesize the target complex.^8^

## Results and Discussion

**Figure 1:**
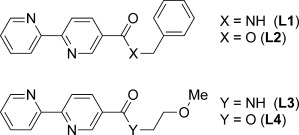
The ligand systems employed in the combinatorial library.

**Identification of appropriate ligand systems**: The primary objective of this study was to demonstrate that a combinatorial library of products can first be appropriately speciated by using routine electrospray spectroscopy, and then demonstrate that the library can be perturbed by the introduction of an external stimulus. Having selected labile iron(II) complexes of 2,2′-bipyridine and armed with the understanding that substitution at the 5-position of the ligands with amidic functions is known to give a relatively strong interaction with electronegative anions,^6^–^8^ we selected chloride as a suitable component to add to the combinatorial library, given its benign nature under normal electrospray conditions assuming that it does not cause either a significant perturbation in the ionic strength or precipitation. The problem with many 2,2′-bipyridine amide-functionalized ligand systems reported for the recognition of anions^44^ using the tris-chelating ruthenium(II) centre is their solubility.^6^, ^45^ To overcome this problem, and the possibility that inter-ligand steric interactions can dominate larger ligand systems around the iron(II) centre, 5-benzylamido-2,2′-pyridine (L1, Figure 1) was selected with the aromatic group separated from the amide by a flexible methylene spacer, and only one functional group per ligand. This ligand system has previously been shown to have a good response to the introduction of anions in the complex *mer-* and *fac*-[Ru(L1)_3_]^2+^.^8^

**Figure 2:**
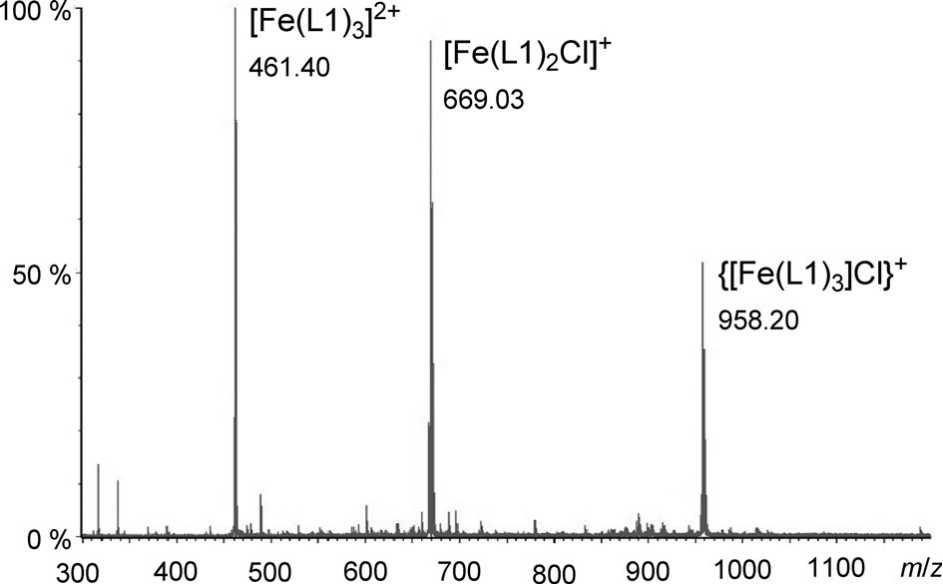
ESI-MS spectrum of ligand L1 with FeCl_2_ in acetonitrile (metal ion concentration=50 μm).

Initial ESI-MS experiments indicated that a relatively straight forward spectrum, typical of the ionization of [Fe(L1)_3_]Cl_2_, could be obtained by taking a 10 μL solution of a 10 mm stock solution of anhydrous FeCl_2_ in methanol and mixing it with 60 μL of a 10 mm stock solution in acetonitrile of L1 and diluting the sample to 50 μm with acetonitrile (see the Experimental Section: Method A). Initially it was hoped to use only acetonitrile, however the solubility of the iron salt dictated that a small quantity of methanol was required to permit the initial dissolution. The detected spectrum (Figure 2) proved to be diagnostic, with signals for both [Fe(L1)_3_]^2+^ at *m/z* 461 and {[Fe(L1)_3_]Cl}^+^ at *m/z* 958 being particularly clear, in addition to peaks representing [L1H]^+^ at *m/z* 290, and {[Fe(L1)_2_]Cl}^+^ at *m/z* 669.

To ensure the iron(II) complex could be formed and detected in situ with structurally similar ligands to L1, the corresponding ester ligand L2 was prepared by using a similar synthetic procedure to that previously reported for the isolation of L1.^8^ The resulting mass spectrum proved to be comparable under the same conditions (see the Supporting Information, Figure S1) with the observation of the doubly charged complex [Fe(L2)_3_]^2+^ at *m/z* 463 and [Fe(L2)_2_Cl]^+^ at *m/z* 671 in addition to peaks representing [L2H]^+^ at *m/z* 291. However, to demonstrate speciation between the ligands, an appropriate soluble 2,2′-bipyridine ligand was required. The criteria were that the ligands should not have significantly different steric or electronic behaviour and noticeably different masses so that the respective molecular ions could be readily realized from the mass spectrum. Initial investigations with 2,2′-bipyridine, 5-methyl-2,2′-bipyridine and 2,2′-bipyridine-4,4′-di(carboxylic acid methyl ester) proved to be unsatisfactory, either due to ligand insolubility, or the dominance of a species attributed to [Fe(L)_2_Cl]^+^ in the resulting spectra. To overcome these problems, the readily soluble amide and ester ligands L3 and L4 were prepared. The mass spectrometry data on these two samples were recorded using an identical procedure as before (Method A, the Supporting Information Figures S2 and S3). For the amide-containing ligand, L3 peaks attributed to the complexes [Fe(L3)_3_]^2+^ at *m/z* 413, {[Fe(L3)_2_]Cl}^+^ at *m/z* 605, {[Fe(L3)_3_]Cl}^+^ at *m/z* 862 and the free ligand [L3H]^+^ at *m/z* 258 were observed, whereas the corresponding ester L4 gave indicative peaks for [L4H]^+^ at *m/z* 259, [Fe(L4)_3_]^2+^ at *m/z* 415 and [Fe(L4)_2_Cl]^+^ at *m/z* 607. Interestingly, neither of the ester ligands L2 and L4 resulted in an assignable signal attributed to {[Fe(L)_3_]Cl}^+^ giving an indication that there is a much higher affinity for the chloride ion with ligand possessing an amide group over the analogous ester functionality.

**Scheme 1:**
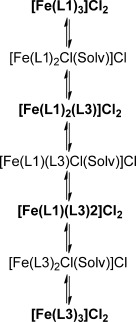
The equilibrium identified within a combination of [Fe(L1)_3_]Cl_2_ and [Fe(L3)_3_]Cl_2_ in acetonitrile.

**Figure 3:**
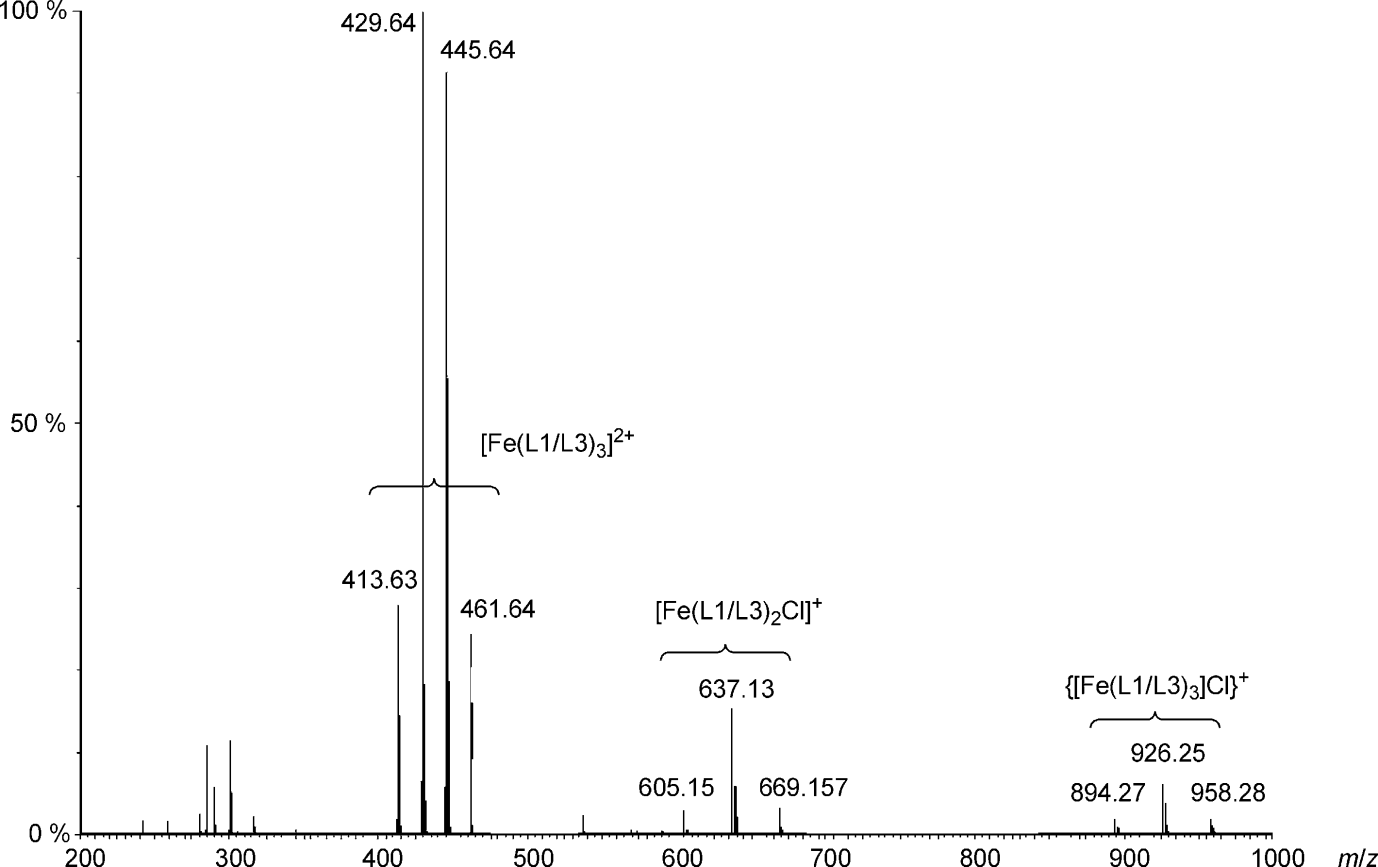
ESI-MS spectrum of ligands L1 and L3 with FeCl_2_ in acetonitrile 30 min after mixing (metal ion concentration=50 μm)

**Figure 4:**
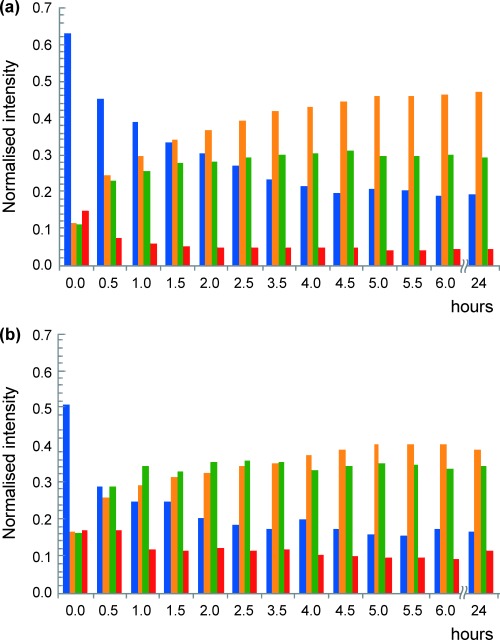
Time course graphs of normalized intensity obtained for FeCl_2_:L1:L3, determined for (a) {[Fe(L1/L3)_3_]Cl}^+^ (*m/z* 862, 894, 926, 958) species and (b) [Fe(L1/L3)_3_]^2+^ (*m/z* 413, 429, 445, 461). [Fe(L1)_3_] (blue), [Fe(L1)_2_(L3)] (orange), [Fe(L1)(L3)_2_] (green) and [Fe(L3)_3_] (red).

**Equilibria in mixed ligand systems**: The two amide-containing ligands L1 and L3 were selected for the initial studies on the composition of heteroleptic solutions given that the individual homoleptic metal complexes gave clean and interpretable mass spectra for the iron(II) complexes. The first step of the investigation was to establish whether the metal complexes were at equilibrium (Scheme 1) under the conditions required to provide interpretable spectra, and significantly whether the species being detected were representative of the solution composition. The two complexes [Fe(L1)_3_]Cl_2_ and [Fe(L3)_3_]Cl_2_ were prepared separately, and then mixed in a 1:1 ratio following the protocol outlined in preparation method A. An excess of both ligands was used (in this case, 6 equivalents) to each metal ion to minimize the presence of the bischelate species [Fe(L)_2_Cl]^+^. The spectra of the resulting pink solutions were then recorded over a period of 24 h establishing the time taken for the complexes to reach equilibrium at 298 K. With anhydrous FeCl_2_, using a little methanol to encourage solubility of the iron salt, the resulting mass spectra recorded after 30 min of mixing the two solutions demonstrated that the key regions of interest could be readily identified with peaks indicative of both bis- and tris-bipyridine species (Figure 3). Clusters of peaks are observed for the exchange complex species for [Fe(L1/L3)_3_]^2+^ at *m/z* 413–461, [Fe(L1/L3)_2_Cl]^+^ at *m/z* 606–669, and {[Fe(L1/L3)_3_]Cl}^+^ at *m/z* 862–958 ({[Fe(L1/L3)_3_]Cl}^+^. Over the course of the experiment, the relative intensities for both the [Fe(L1)/(L3)_3_]^2+^ (*m/z* 413, 429, 445, 461) and {[Fe(L1)/(L3)_3_]Cl}^+^ (*m/z* 862, 894, 926, 958) species were normalized and their relative change over time plotted (Figure 4). This simple system appears to follow first order kinetics (*k*_Cl_=(1.7±0.1×10^−4^) s^−1^), with the system reaching equilibrium in the order of four hours demonstrating the anticipated speciation in the final products. Importantly the two species investigated, the divalent ion [Fe(L)_3_]^2+^ and the monovalent ion {[Fe(L)_3_]Cl}^+^, gave similar time plots despite having different relative intensities in the spectra analysed. Interestingly, the complexes formed from ligand L1 appears to be the more dominant over those composed of L3. Initially it was considered if this observation arose from the two different complexes having dissimilar ionization behaviour, resulting in lower detection of the species containing ligand L3, however the ion count for the complexes [Fe(L1)_3_]^2+^ and [Fe(L3)_3_]^2+^ under similar conditions were comparable indicating that the observed speciation in the heteroleptic system is probably representative of the composition of the solution, with ligand L1 having a greater thermodynamic stability on the metal centre than L3. One would have expected the more sterically demanding ligand to have shown the weaker relative binding but that does not appear to be the case.

The length of time taken for the system to achieve equilibrium was a surprising observation; in our preliminary studies it was assumed that in acetonitrile a mixture of an iron(II) salt and bipyridine ligands rapidly reached equilibrium given the instantaneous development of the characteristic dark pink colour upon mixing. This is consistent with a relatively quick formation of the kinetic product with the free metal ions. The subsequent ligand substitution, necessitating the displacement of a chelating bipyridine, to arrive at the thermodynamic distribution is much slower. This latter kinetic process determines the distribution of the dynamic library and so needs to be taken into consideration when exploring the subsequent perturbation of an observed mixture of complexes.

The study was extended to a range of other iron(II) salts to investigate the effect that the counteranion has on the time taken for the system to reach equilibrium. The use of FeBr_2_ as the iron source resulted in very similar behaviour to that observed for FeCl_2_ (the Supporting Information, Figure S4; *k*_Br_=(1.6±0.1×10^−4^) s^−1^) with a slightly longer time to come to equilibrium (approx. five hours). In moving to iron(II) sources with anions that have a lower affinity to metal coordination, namely Fe(ClO_4_)_2_ and Fe(BF_4_)_2_, the time required to come to equilibrium was considerably longer (the Supporting Information, Figures S5 and S6; rate constants of *k*_CLO__4_ =(0.82±0.05×10^−4^) s^−1^ and *k*_BF__4_ = (0.54±0.05×10^−4^) s^−1^ respectively), and was only fully complete after 24 h in the latter case. This indicates that the nature of the counterion is extremely important in the process, with anions that readily coordinate to the metal ions making the exchange process occur more rapidly. Studies were also attempted using FeSO_4_, but precipitation of the induced complexes were observed approximately ten minutes after sample preparation and so no further investigation was undertaken with this system.

**Figure 5:**
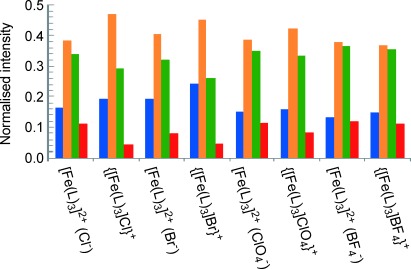
Normalized intensities obtained for FeX_2_/L1 L3, determined after 24 h for {[Fe(L1/L3)_3_]X}^+^ and [Fe(L1/L3)_3_]^2+^. [Fe(L1)_3_] (blue), [Fe(L1)_2_(L3)] (orange), [Fe(L1)(L3)_2_] (green) and [Fe(L3)_3_] (red).

Comparison of the final distribution of the four complexes observed in the mass spectrum with the four salts tested after 24 h indicates that for the divalent species [Fe(L)_3_]^2+^, the counteranion does not significantly affect the speciation (Figure 5), which approaches the expected statistical polynomial distribution (1:3:3:1) of the four species (with a slight preference for ligand L1 over L3 as previously observed). A remarkably similar distribution was also observed in the monovalent species containing either perchlorate or tetrafluoroborates ions. However, in the presence of simple halide ions, the preference for L1 over L3 in the complex {[Fe(L1)_3_]X}^2+^ (in which X is Cl or Br) is further exaggerated giving an indication that a bound anion can determine the product distribution. Hence, the anion is not only involved in the rate at which the system reaches equilibrium, but significantly, it is also involved in determining the equilibrium, as would be anticipated if these anions are “binding” to the amide ligand systems.

The speciation of the mixtures resulting from pairs of both the two ester ligands (L2 and L4) and then with the amides (L1 and L3), using pre-prepared homoleptic samples (method A) in the presence of FeCl_2_, FeBr_2_, Fe(ClO_4_)_2_ and Fe(BF_4_)_2_, were also considered both shortly after mixing, and then 24 h later. In each case, the iron(II) halide salts resulted in a mixture that was evidently a combination of all four possible components shortly after mixing the two components, but there were significant differences with the spectra taken after 24 h (Figure S7–S9). The perchlorate and tetrafluoroborates salts were again observed to take considerably longer to reach equilibrium than the halide salts following the order: Cl (shortest)<Br<ClO_4_<BF_4_ (longest).

**Figure 6:**
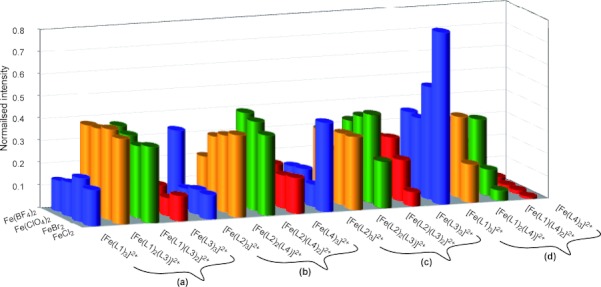
Normalized intensities of obtained complexes determined after 24 h for [Fe(L^A^/L^B^)_3_]^2+^ after mixing two solutions of the iron(II) source in the presence of three ligands of (a) L1 and L3, (b) L2 and L4, (c) L2 and L3 and (d) L1 and L4 (metal ion concentration 50 μm). [Fe(L^A^)_3_] (blue), [Fe(L^A^)_2_(L^B^)] (orange), [Fe(L^A^)(L^B^)_2_] (green) and [Fe(L^B^)_3_] (red).

The final distribution of the divalent cations [Fe(L)_3_]^2+^ developed from the ligand combinations L2 and L4, L2 and L3, and L1 and L4 after 24 h of equilibration also provided informative data (Figure 6). In the majority of ligand combinations with the counter tetrafluoroborates and perchlorate salts, the distribution of products proved to be similar to that seen with L1 and L3, which is consistent with equilibrium having been achieved in all but one case; the distribution for the ester combination of L2 and L4 with Fe(BF_4_)_2_ was still dominated by peaks assigned to [Fe(L2)_3_]^2+^ and [Fe(L4)_3_]^2+^. The ligand combination L2 with L3 in the presence of FeCl_2_ does not follow the expected 1:3:3:1 distribution of [Fe(L2)_3_]^2+^, [Fe(L2)_2_(L3)]^2+^, [Fe(L2)(L3)_2_]^2+^, and [Fe(L3)_3_]^2+^, with the observed dominance of [Fe(L2)_3_]^2+^ although it would appear that in this case the distribution of products has come to equilibrium given the depletion of [Fe(L3)_3_]^2+^ from the system. This is further exaggerated with ligands L1 and L4, with complexation of the amide L1 dominating the coordination of the metal centre, with the inference being that the chloride anion “templates” the formation of the homoleptic amide species, presumably through the hydrogen bonding interactions of the amidic protons with the chloride in keeping with the findings of the previously reported ruthenium(II) complexes.^8^ This affect is also observed with the iron(II) complexes formed from FeBr_2_ and the L1 and L4 ligand system, but to a lesser extent.

Given the observations, by mixing preformed complexes and letting them come to equilibrium, there is strong evidence that ESI-MS can be used to both understand the time to reach equilibrium, and the eventual speciation with reasonable certainty as to the identity of the dominant species, with the data being consistent with the results we have previously obtained for analogous ruthenium(II) complexes.^8^

**Figure 7:**
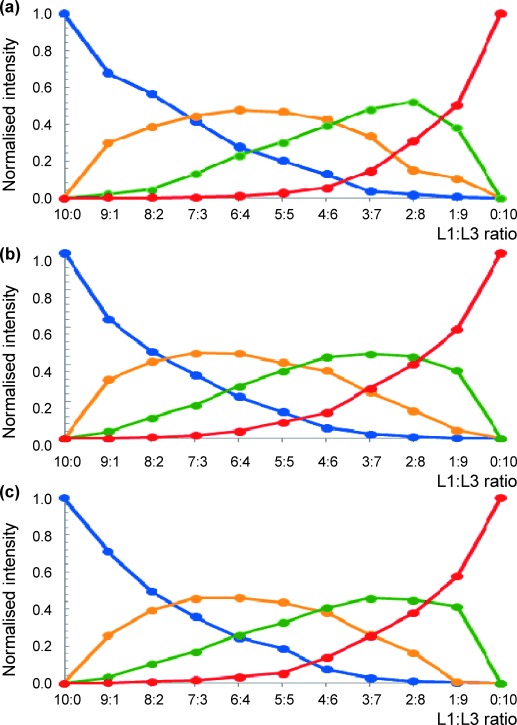
Speciation plots of [Fe(L1/L3)_3_]^2+^ determined from the mass spectroscopy data with varying concentrations of ligands L1 and L3 in the presence of (a) FeCl_2_, (b) Fe(ClO_4_)_2_ and (c) Fe(BF_4_)_2_. [Fe(L1)_3_] (blue), [Fe(L1)_2_(L3)] (orange), [Fe(L1)(L3)_2_] (green) and [Fe(L3)_3_] (red).

**Speciation in mixed ligand systems**: To see if the relative ratios of the complexes following electrospray ionization are typical of those that would be anticipated in solution, and in the absence of any other technique available to us to determine the nature of the solution composition, the interaction of ligands L1 and L3 with the iron(II) salts was also studied by premixing the two ligands prior to the addition of the metal cations. A total of 10 molar equivalents of the ligands were used rather than 6 (see the Experimental Section: Method B) so that depletion of one of the ligands would not significantly affect the overall speciation, but without becoming sufficiently large that the ligands themselves dominate the spectra. The ligand ratio was systematically varied from 100 % L1 to 100 % L3 in 10 % incremental steps using a traditional Job plot analysis. Using FeCl_2_, FeClO_4_ and FeBF_4_ as the iron(II) sources, there was good evidence of the formation of the four possible tris-chelate bipyridine complexes whose overall concentration is dependent on the ligand ratio used (sample spectra available in the Supporting Information, Figure S10). The relative intensities of the peaks corresponding to the four tris-chelate species recorded after one hour following the addition of the iron salt for both {[Fe(L1/L3)_3_]X}^+^ and [Fe(L1/L3)_3_]^2^ species as determined (Figure 7). The mass spectra were also recorded again after 24 h following the initial sample preparation, and comparison of this spectra with the spectra recorded after 1 hour revealed that the relative composition was invariant over this period. It would appear that under these conditions, the anion itself does not significantly affect the overall equilibrium, although the evidence indicates that, as with the previously discussed experiments, there is a slight preference for complexes containing ligand L1 over L3, which is marginally exaggerated in the presence of a chloride anion (see the Supporting Information, Figure S11). Using a premixed ligand system seems to ensure that the thermodynamic mixture of products is isolated in a relatively short time given that the initial kinetic mixture is close to the final thermodynamic distribution.

The combination of the ester containing ligands L2 and L4 was also studied by premixing the ligands prior to addition of the iron source again using FeCl_2_, FeBr_2_, Fe(ClO_4_)_2_ and Fe(BF_4_)_2_ as the iron sources. Again there is evidence of the formation of the four possible metal complexes with the relative ratio of the species dependent on the ligand ratio used and again similar mass spectra were obtained with all iron(II) sources (the Supporting Information, Figure S12). It would appear that under these conditions, once again the anion present from the iron salt does not appear to greatly affect the overall equilibrium with no significant preference for either of the two ligands.

The interaction of the ester/amide ligand combinations L2/L3, and L4/L1 with Fe^2+^ was also studied using a pseudo-Job plot analysis using Fe(BF_4_)_2_ as the iron(II) salt. Problems were encountered using FeCl_2_ as the source of the metal cation with these ligand systems, disappointingly resulting in very low total ion counts and correspondingly unusable spectra. This could have arisen either from the presence of a slight precipitation, or due to the possible interaction of the amide ligands with the chloride anions which significantly disturbed the equilibrium. In considering the L2/L3 system with Fe(BF_4_)_2_, there is evidence of the formation of the four possible interchanged tris-chelate bipyridine complexes whose concentrations are generally proportional to the ligand ratio used. The relative intensities again were calculated and a distribution plot for both the [Fe(L)_3_]^2+^ and {[Fe(L)_3_](BF_4_)}^+^ were considered (the Supporting Information, Figure S13). In this case, there is a slight preferential binding for the amidic ligand L3 over the ester containing L2, particularly in the 2+ charge species. The corresponding L4/L1 system was not particularly easy to analyse, with what appears to be poor data for the 9:1 and 3:7 ratios (the Supporting Information, Figure S14), but despite this, it would appear that the amide ligand (L1) would also be marginally preferred over L4.

On the evidence available from these speciation plots, the relative peak intensities of the four species present in the spectra are representative of the anticipated speciation in the solution, with a preference for the amide ligands systems over those of the ester functions, presumably due to the opportunities for additional hydrogen bonding, whereas the benzyl-containing species are preferred over the methoxyethyl group. These results, using premixed species (in a 5:5 ratio), replicate the equilibrium achieved after mixing the individual complexes and as a consequence, it is a reasonable assumption that the observed relative intensities determined by ESI-MS do appear to be directly related to the anticipated solution composition.

**Perturbation of the equilibrium**: Having established that the distribution of the complexes in solution could be determined by ESI-MS, and that the ligand systems under investigation were under a dynamic equilibrium, the next consideration was whether introducing an external stimuli could cause a shift in the distribution. The above results have demonstrated that the equilibrium, particularly that of the {[Fe(L)_3_](X)}^+^ species, is dependent on the anion present. Similarly, our preceding studies have also shown that the kinetically inert species containing ligand L1 on ruthenium(II) have a degree of selectivity for dihydrogen phosphate and chloride over a range of other anions.^8^ The introduction of phosphate ions is problematic given the propensity for complexes of this type to precipitate and for phosphate to hinder ionisation. As a result, the addition of chloride ions was considered to the iron(II) salts of the complexes formed from a 5:5 ratio of pairs of ligand systems using Fe(BF_4_)_2_ as the metal cation source, making the assumption that the BF_4_ anion has a negligible coordination to either the ligands or metal ions involved. Ammonium chloride was initially selected as the salt of choice, with the ammonium cation being far from the mass region of interest; however, it also proved to be problematic because it gave an unidentified precipitate. The corresponding tetrabutylammonium (TBA) salt however proved to give reasonable spectra of the regions of interest with the addition of up to ten equivalents of the anions concerned, although slight problems were encountered arising from the increased ion count.

**Figure 8:**
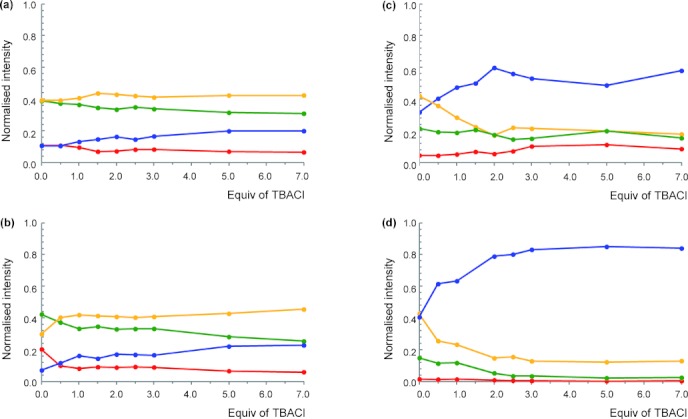
Speciation plots of [Fe(L^A^/L^B^)_3_]^2+^ using premixed equimolar quantities of (a) L1 and L3, (b) L2 and L4, (c) L3 and L2 and (d) L1 and L4, with Fe(BF_4_)_2_ in the presence of increasing equivalents of tetrabutylammonium chloride after 1 hour equilibrium (acetonitrile as solvent), metal ion concentration 50 μm. [Fe(L^A^)_3_] (blue), [Fe(L^A^)_2_(L^B^)] (orange), [Fe(L^A^)(L^B^)_2_] (green) and [Fe(L^B^)_3_] (red).

On addition of TBACl to a distribution arising from equimolar ligand combinations and Fe(BF_4_)_2_ there were clear differences observed depending on the ligand combination used. In case of the amide ligand system (L1/L3), a slight change in the distribution of the species present was observed; with increasing amounts chloride present, there is an increased quantity of [Fe(L1)_3_]^2+^ and a corresponding decrease in [Fe(L1)(L3)_2_]^2+^ and [Fe(L3)_3_]^2+^ giving an indication that the complexes formed using L1 have a marginally greater preference for chloride than those composed from ligand L3 (Figure 8 a). Similar behaviour was shown by the ester ligand system (L2/L4), with a slight preference for the stabilization of metal complexes containing ligand L2 bearing the benzylester group (Figure 8 b) on the addition of chloride. It can be assumed therefore that the anions must be weakly associated with the aromatic functionality present in both L1 and L2. However for both of these systems, these effects are small, and could potentially be dismissed as being insignificant.

For the mixed amide/ester ligand systems, the titrations demonstrated very different behaviour and clearly illustrate the preference for amidic functionality in the metal complexes in the presence of large quantities of halide ions (Figure 8 c and d). The spectra for the L2/L3 system (the Supporting Information, Figure S15 a) shows that upon addition of TBACl, the peak corresponding to [Fe(L3)_3_]^2+^ (*m/z* 415) has increased considerably at the expense of higher weight species containing the benzyl ester L2, whereas the amide containing complexes are formed preferentially over the corresponding esters (as discussed previously). The introduction of chloride clearly drives this equilibrium further as demonstrated by the depletion of [Fe(L2)(L3)_2_]^2+^. This is evident to an even greater extent in the L1/L4 mixture, with the peak corresponding to [Fe(L1)_3_]^2+^ (*m/z* 461) increasing at the expense of complexes containing L4 (the Supporting Information, Figure S15 b). In addition to the dominance of the amide containing species, the affect of the benzyl group further enhances the observed preference.

Given the titration data, the evidence shows that the benzylamide derivative (L1) does appear to be the overall preferred ligand for use in the formation of complexes to bind to chloride, with ESI-MS demonstrating the suppression of similar species. On the addition of chloride to the mixtures, a colour change was also observed with the solutions becoming darker (more purple). This observation is readily monitored by UV/Vis spectroscopy with a systematic change being observed on the sequential addition of TBA chloride to a mixture of Fe(BF_4_)_2_ and five equivalents of both L1 and L4 (the Supporting Information, Figure S17). However, given that four complexes are contributing to the observed metal-to-ligand-change-transfer band (not including species with metal bound chloride ions), multivariable modeling to obtain meaningful speciation data would be required even for this simple two ligand system. Attempts were also made to determine if a similar change in speciation could be determined by ^1^H NMR spectroscopy. However, severe difficulties were observed; very broad peaks were encountered, either due to the presence of a small percentage of paramagnetic iron salts, and/or the dynamic nature of the system. Given the complexity of the large number of overlapping signals in the ^1^H NMR spectra, integration of characteristic regions was not possible. The use of ESI-MS does however give direct access to the species present, where it would appear that both the gas and liquid phase studies are sufficiently similar to permit determination of the dominant species.

## Conclusion

In the course of this work, an ESI-MS protocol has been established making it possible to evaluate the equilibrium of different bipyridine derivatives around an iron(II) centre, through the premixing of ligands followed by the introduction of an iron(II) salt. The resulting dynamic equilibrium has then been shown to be perturbed by the addition of an external stimulus, in this case chloride. As indicated in the introduction, the design of anion receptors has over the years required considerable synthesis to create a suite of compounds to be directly compared to optimize for a degree of selectivity, certainly in our experience with ruthenium(II) complexes this has proved to be a considerable challenge. The use of a dynamic combinatorial library centred on iron(II) and directly interrogated by ESI-MS has been shown here to have opportunities to identify potential candidates for a particular target analyte without undertaking considerable and potentially redundant synthetic work. However it is acknowledged that there are certain considerations that need to be made in creating a working model system. In this paper we have demonstrated that using components that are sufficiently similar having analogous ionization potentials, that “fly” in the instrument together, and with comparable detection responses, these concerns can be mitigated. We have also confirmed that the resulting distribution of the iron complexes in the gas phase corresponds to that anticipated in the liquid phase.

Given the evidence outlined in this report, we conclude that the experimental protocol described herein has the potential to be an extremely powerful tool in the identification of synthetic target structures for the recognition of a wide variety of analytes. Given the continuing widespread consideration of ruthenium(II) polypyridyl complexes in the detection of DNA,^2^ protein,^3^, ^46^ and small molecules,^1^ and the difficulty in the synthetic procedures of these inert complexes, the initial screening using an iron(II) dynamic library monitored by ESI-MS will remove the requirement for a considerable amount of protracted and difficult synthesis. At the current time, the groups involved in this study are extending the ideas shown to the design of recognition units for protein surfaces and key structural features in DNA.

## Experimental Section

**Instrumentation**: ^1^H and ^13^C NMR spectra were recorded on a Bruker AV300, microanalyses and E.I. mass spectrometry were performed by A.S.E.P., The School of Chemistry, The Queen’s University of Belfast. Electrospray mass spectra were recorded on a LCT Premier electrospray mass spectrometer (Waters) fitted with a Nanomate injection system (Advion). Analysis was completed by using 10 μL samples with an acquisition time of 1 min. The calibration of the spectrometer was checked using SULPHA prior to each acquisition set, with the capillary voltage set at 4229 V, cone voltage at 100 V and the source temperature at 120 °C, respectively. Data analysis was completed using Mass Lynx v4.1, using both isotope modeling and elemental composition to confirm the proposed species where possible. Samples were analysed in positive mode using standard ESI+ acquisition parameters whilst checking the total ion count (typically between 10^4^–10^6^). If the ion count value exceeded 10^6^, the solution was diluted with acetonitrile and re-run.

**Materials**: Anhydrous FeCl_2_, Fe(ClO_4_)_2_**⋅***x*H_2_O and Fe(BF_4_)_2_**⋅**6H_2_O were purchased from Sigma Aldrich, 2,2′-bipyridine was purchased from Alfa Aesar. HPLC grade methanol and acetonitrile were used throughout. THF was dried by distillation from sodium under nitrogen. 2,2′-Bipyridine-5-carboxylic acid^47^ and 5-benzylamido-2,2′-bipyridine (L1)^8^ were prepared following literature procedures.

**Synthesis**: All ligands were prepared by a similar route (see below).

*2,2′-Bipyridine-5-carboxylic acid benzylester (**L2**)*: Dry 2,2′-bipyridine-5-carboxylic acid (1.00 g, 5.00 mmol) was refluxed in thionyl chloride (25 cm^3^) under nitrogen for 3 h, giving a clear yellow solution. The thionyl chloride was removed in vacuo and the residual solid dried under vacuum for 1 h. The acyl chloride was dissolved in dry THF under nitrogen (50 mL) and brought to reflux. To this, a solution of benzyl alcohol (0.52 mL; 5.00 mmol) dissolved in dry THF (5 mL) and triethylamine (2 mL) was added dropwise over 1 h, and the resulting mixture cooled and stirred for 16 h at room temperature. This was poured into water (50 mL) and extracted using CH_2_Cl_2_ (2×50 mL). The organic layer was dried over anhydrous MgSO_4_, filtered and the solvent removed under reduced pressure leaving a light brown solid. The crude product was dissolved in diethyl ether (50 mL), washed with water (2×50 mL) and dried over anhydrous MgSO_4_ to afford the product as a pale yellow/brown solid (0.507 g, 35 %). ^1^H NMR (300 MHz, CDCl_3_): *δ*=5.42 (s, 2 H; CH_2_), 7.32–7.51 (m, 6 H; H^ar^+H^bpy5′^), 7.85 (dd, *J*=7.5 Hz and 8.1 Hz, 1 H; H^bpy4′^), 8.43 (d, *J*=8.3 Hz, 1 H; H^bpy4^), 8.48 (d, *J*=8.1 Hz, 1 H; H^bpy3′^), 8.50 (d, *J*=8.3 Hz, 1 H; H^bpy3^), 8.71 (d, *J*=4.8 Hz, 1 H; H^bpy6′^), 9.31 ppm (s, 1 H; H^bpy6^); ^13^C NMR (75.47 MHz, CDCl_3_): *δ*=67.1, 120.5, 121.9, 124.5, 125.6 (q), 128.3, 128.4, 128.7 135.6 (q) 137.0, 138.1, 149.4, 150.6, 155.5 (q), 158.9 (q), 165.2 ppm (q); EI-MS *m/z* calcd for C_18_H_14_N_2_O_2_: 290.1055 [*M*]^+^; found: 290.1060; elemental analysis calcd (%) for C_18_H_14_N_2_O_2_: C, 74.47, H, 4.81, N, 9.65; found: C, 74.43, H, 4.71, N, 9.46.

*5-(2-Methoxyethane)amido-2,2′-bipyridine (**L3**)*: Prepared using the same procedure used to isolate L2 with the addition of 2-methoxyethylamine rather than benzylalcohol (0.76 g, 56 %). ^1^H NMR (300 MHz, CDCl_3_): *δ*=3.39 (3 H, s, CH_3_), 3.58 (2 H, t, *J*=5.0 Hz, CH_2_), 3.68 (dt, *J*=5.0 Hz and 5.0 Hz, 2 H; CH_2_), 6.80 (br, 1 H; NH), 7.33 (dd, *J*=4.8 Hz and 7.5 Hz, 1 H; H^bpy5′^), 7.82 (dd, *J*=7.5 and 7.8 Hz, 1 H; H^bpy4′^), 8.19 (d, *J*=8.3 Hz, 1 H; H^bpy4^), 8.44 (d, *J*=7.8 Hz, 1 H; H^bpy3′^), 8.47 (d, *J*=8.3 Hz, 1 H; H^bpy3^), 8.68 (d, *J*=4.8 Hz, 1 H; H^bpy6′^), 9.06 ppm (s, 1 H; H^bpy6^); ^13^C NMR (75.47 MHz, CDCl_3_): *δ*=40.2, 59.2, 71.4, 121.0, 122.0, 124.7, 130.1 (q), 136.2, 137.4, 148.2, 149.7, 155.5 (q), 158.9 (q), 166.0 ppm (q); EI-MS: *m/z*: calcd for C_14_H_15_N_3_O_2_: 257.1164 [*M*]^+^ ; found: 257.1169; elemental analysis calcd (%) for C_14_H_15_N_3_O_2_: C, 65.36, H, 5.88, N, 16.33; found: C, 65.20 H, 5.74 N, 16.36.

*2,2′-Bipyridine-5-carboxylic acid 2-methoxyethane ester (**L4**)*: prepared using the same procedure used to isolate L2 with the addition of 2-methoxyethanol rather than benzylalcohol (0.465 g, 36 %). ^1^H NMR (300 MHz, CDCl_3_): *δ*=3.44 (s, 3 H; CH_3_), 3.75 (t, *J*=4.6 Hz, 2 H; CH_2_), 3.68 (d, *J*=4.6 Hz, 2 H; CH_2_), 7.35 (dd, *J*=4.9 Hz and 7.4 Hz, 1 H; H^bpy5′^), 7.84 (dd, *J*=7.4 and 7.9 Hz, 1 H; H^bpy4′^), 8.42 (d, *J*=8.3 Hz, 1 H; H^bpy4^), 8.47 (d, *J*=7.9 Hz, 1 H; H^bpy3′^), 8.50 (d, *J*=8.3 Hz, 1 H; H^bpy3^), 8.70 (d, *J*=4.9 Hz, 1 H; H^bpy6′^), 9.29 ppm (s, 1 H; H^bpy6^); ^13^C NMR (75.47 MHz, CDCl_3_): *δ*=59.1, 64.4, 70.4, 120.4, 121.9, 124.5, 125.5 (q), 137.0, 138.1, 149.3, 150.6 155.0 (q), 159.5 (q), 165.3 ppm (q); EI-MS: *m/z*: calcd for C_14_H_14_N_2_O_3_: 258.1004 [*M*]^+^; found: ) 258.1003 ; elemental analysis calcd (%) for C_14_H_14_N_2_O_3_: C, 65.11, H, 5.46, N, 10. 85; found: C, 65.22 H, 5.30, N, 11.05.

**ESI-MS sample preparation**: 10 mm stock solutions of the ligands and the iron salts were prepared in acetonitrile, with the exception of FeCl_2_, which was prepared in methanol.

*Method A*: In an Eppendorf tube, the ligand (60 μL) and the appropriate iron salt stock solution (10 μL) were mixed. The solution was diluted to an overall concentration of 50 μm by taking 70.4 μL of this solution and diluting it to 200 μL with acetonitrile). In mixed ligand systems, a 1:1 ratio mixture was obtained by taking 30 μL of each of the diluted iron(II) solutions in a separate Eppendorf tube.

*Method B*: Using 10 μL as being one equivalent, the appropriate number of equivalents required giving an overall total volume of 100 μL of the mixed ligand system (i.e., 10 equivalents) were placed in an Eppendorf tube. A series of 11 samples ranging from 0:10 to 10:0 ligand ratios were typically prepared. Then 10 μL of the iron salt stock solution was added and the sample left for 1 h to come to equilibrium, before recording the spectra, which was then re-recorded after 24 h to ensure consistency.

*Addition of TBACl to the MS samples*: 10 mm and 100 mm stock solutions of tetrabutylammonium chloride (TBACl) were prepared in acetonitrile. The appropriate volume of the TBACl stock solutions were used to add 0 to 10 equivalents to 50 μL of the stock solutions of the two ligand systems under investigation, followed by 10 μL of the Fe(BF_4_)_2_ stock solution. The solution was diluted to reach an overall concentration of approximately 50 μm with respect to the iron salt by taking 11 μL of this solution and diluting it to 200 μL with acetonitrile.

**ESI-MS data normalization**: ESI-MS data for systems containing more than one ligand by summation of the peak heights for the four possible components and then reporting the relative intensities of each of the components as a fraction of the total intensity.

## Notes

Detailed facts of importance to specialist readers are published as ”Supporting Information”. Such documents are peer-reviewed, but not copy-edited or typeset. They are made available as submitted by the authors.
