# Supplementary material for: The Use of Electrospray Mass Spectrometry to Determine Speciation in a Dynamic Combinatorial Library for Anion Recognition
Source: Chemistry. 2012 Sep 20;18(43):13733–42. doi: 10.1002/chem.201201302 (PMC3569615; doi:10.1002/chem.201201302)
Supplement: Supplementary file 1 [file chem0018-13733-sd1.pdf]

CHEMISTRY
A EUROPEAN JOURNAL

Supporting Information

© Copyright Wiley-VCH Verlag GmbH & Co. KGaA, 69451 Weinheim, 2012

The Use of Electrospray Mass Spectrometry to Determine Speciation in a Dynamic Combinatorial Library for Anion Recognition

Hazel I. A. Phillips,[a] Aleksey V. Chernikov,[a] Nicholas C. Fletcher,*[a] Alison E. Ashcroft,[b] James R. Ault,[b] Maria H. Filby,[c] and Andrew J. Wilson[b, c]

chem_201201302_sm_miscellaneous_information.pdf

Contents:

Supplementary Figure 1. ESI-MS spectrum of ligand L2 with $FeCl_2$.

Supplementary Figure 2. ESI-MS spectrum of ligand L3 with $FeCl_2$.

Supplementary Figure 3. ESI-MS spectrum of ligand L4 with $FeCl_2$.

Supplementary Figure 4. Time course graphs of normalized intensity obtained for $FeBr_2$:L1:L3, determined for a) $\{[Fe(L1/L3)_3]Br\}^+$ species and b) $[Fe(L1/L3)_3]^{2+}$.

Supplementary Figure 5. Time course graphs of normalized intensity obtained for $Fe(ClO_4)_2$:L1:L3, determined for a) $\{[Fe(L1/L3)_3]ClO_4\}^+$ species and b) $[Fe(L1/L3)_3]^{2+}$

Supplementary Figure 6. Time course graphs of normalized intensity obtained for $Fe(BF_4)_2$:L1:L3, determined for a) $\{[Fe(L1/L3)_3]BF_4\}^+$ species and b) $[Fe(L1/L3)_3]^{2+}$

Supplementary Figure 7. ESI-MS spectra of ligands L2 and L4 with (a) $FeCl_2$, (b) $FeBr_2$, (c) $Fe(ClO_4)_2$ and (d) $Fe(BF_4)_2$ after (i) 24 hours and (ii) 5 minutes

Supplementary Figure 8. ESI-MS spectra of ligands L2 and L3 with (a) $FeCl_2$, (b) $FeBr_2$, (c) $Fe(ClO_4)_2$ and (d) $Fe(BF_4)_2$ after (i) 24 hours and (ii) 5 minutes

Supplementary Figure 9. ESI-MS spectra of ligands L1 and L4 with (a) $FeCl_2$, (b) $FeBr_2$, (c) $Fe(ClO_4)_2$ and (d) $Fe(BF_4)_2$ after (i) 24 hours and (ii) 5 minutes

Supplementary Figure 10. ESI-MS spectra of premixed ligands L1 and L3 with $FeCl_2$ in (a) a 10:0, (b) a 5:5 and (c) a 0:10 ratio after 1 hour

Supplementary Figure 11. Speciation plots of $\{[Fe(L1/L3)_3]X\}^+$ determined from the mass spectroscopy data with varying concentrations of ligands L1 and L3 in the presence of (a) $FeCl_2$, (b) $Fe(ClO_4)_2$ and (c) $Fe(BF_4)_2$

Supplementary Figure 12. Speciation plots of $[Fe(L2/L4)_3]^{2+}$ determined from the mass spectroscopy data with varying concentrations of ligands L1 and L3 in the presence of (a) $FeCl_2$, (b) $FeBr_2$, (c) $Fe(ClO_4)_2$ and (d) $Fe(BF_4)_2$

Supplementary Figure 13. Speciation plots of (a) $[Fe(L2/L3)_3]^{2+}$ and (b) $\{[Fe(L2/L3)_3]BF_4\}^+$ determined from the mass spectroscopy data with varying concentrations of ligands L2 and L3 in the presence of $Fe(BF_4)_2$

Supplementary Figure 14. Speciation plots of (a) $[Fe(L4/L1)_3]^{2+}$ and (b) $\{[Fe(L4/L1)_3]BF_4\}^+$ determined from the mass spectroscopy data with varying concentrations of ligands L4 and L1 in the presence of $Fe(BF_4)_2$

Supplementary Figure 15. ESI-MS spectra of premixed equimolar quantities of ligands L2 and L3 with $Fe(BF_4)_2$ in (a) the presence of 10 equivalents of tetrabutylammonium chloride and (b) without tetrabutylammonium chloride.

Supplementary Figure 16. ESI-MS spectra of premixed equimolar quantities of ligands L1 and L4 with $Fe(BF_4)_2$ in (a) the presence of 10 equivalents of tetrabutylammonium chloride and (b) without tetrabutylammonium chloride.

Supplementary Figure 17. UV / vis spectrum spectra of premixed equimolar quantities of ligands L1 and L4 (5×10^{-4} mol L^{-1}) with $Fe(BF_4)_2$ (1×10^{-4} mol L^{-1}) in (a) the presence of increasing equivalents of tetrabutylammonium chloride (0 to 1×10^{-3} mol L^{-1}) and after 1 hour equilibrium (acetonitrile as solvent).

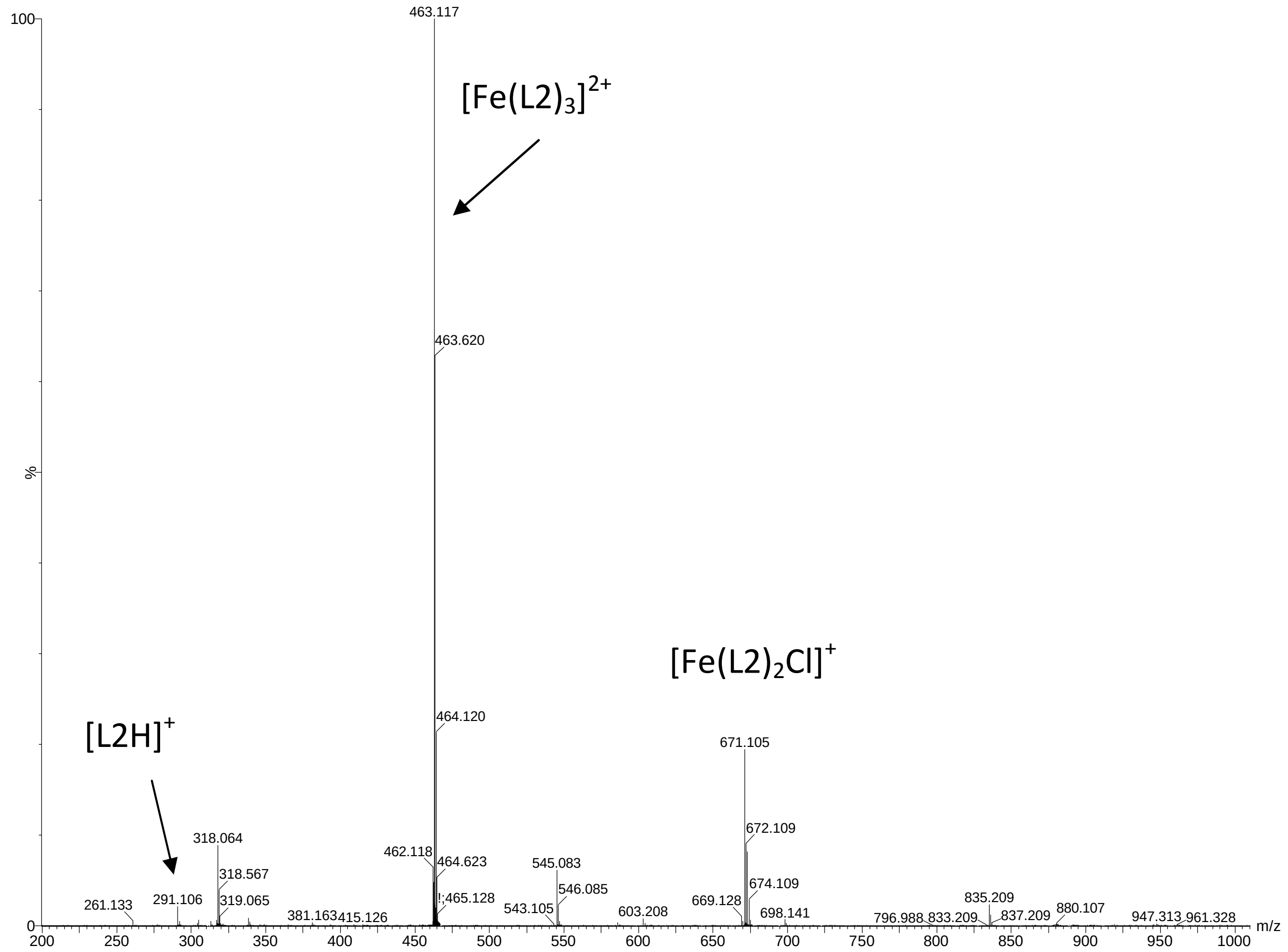

Supplementary Figure 1. ESI-MS spectrum of ligand L2 with $FeCl_2$ (acetonitrile as solvent), metal ion concentration 50 µmol L^{-1}.

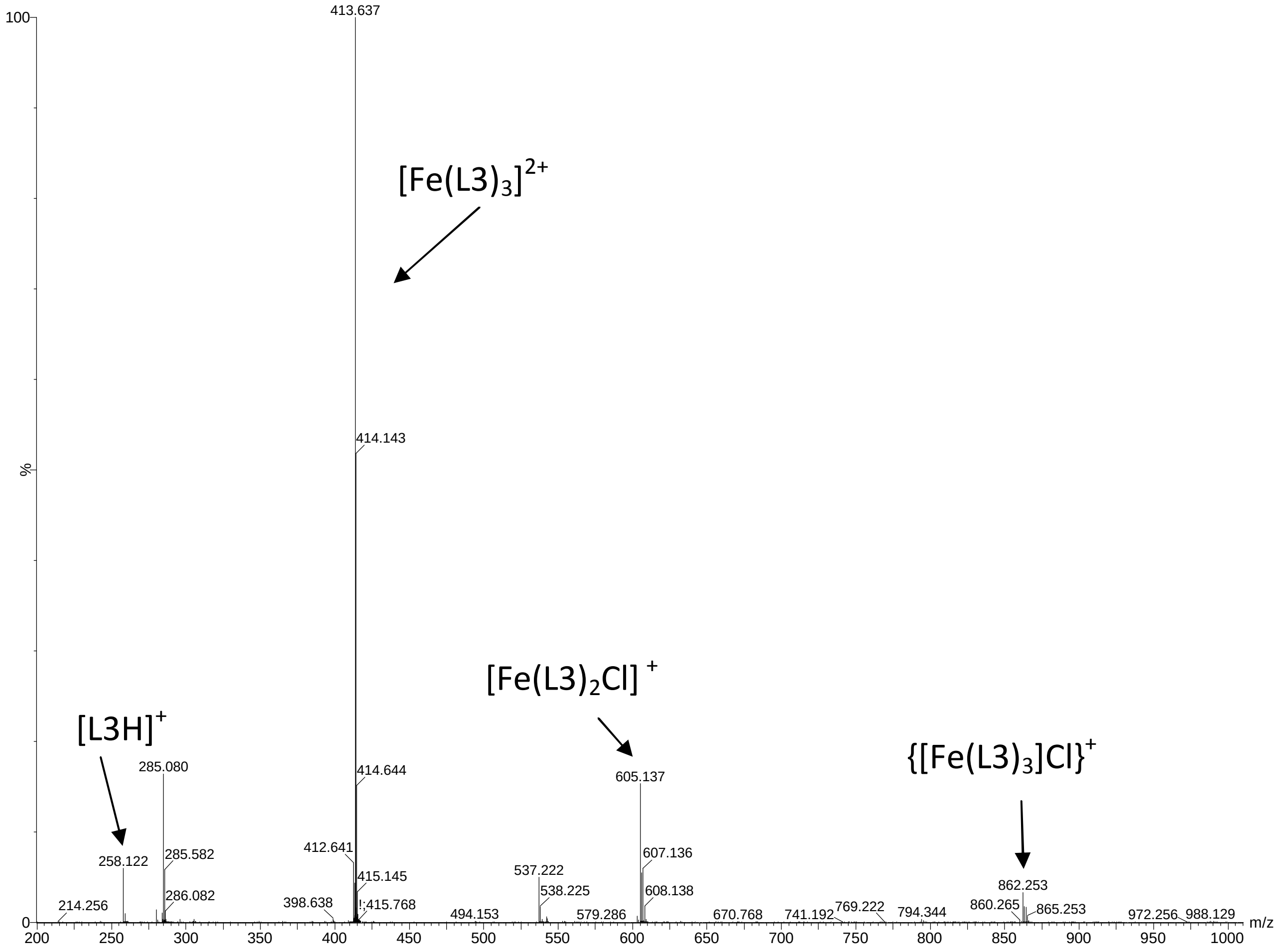

Supplementary Figure 2. ESI-MS spectrum of ligand L3 with $FeCl_2$ (acetonitrile as solvent), metal ion concentration 50 µmol L^{-1}.

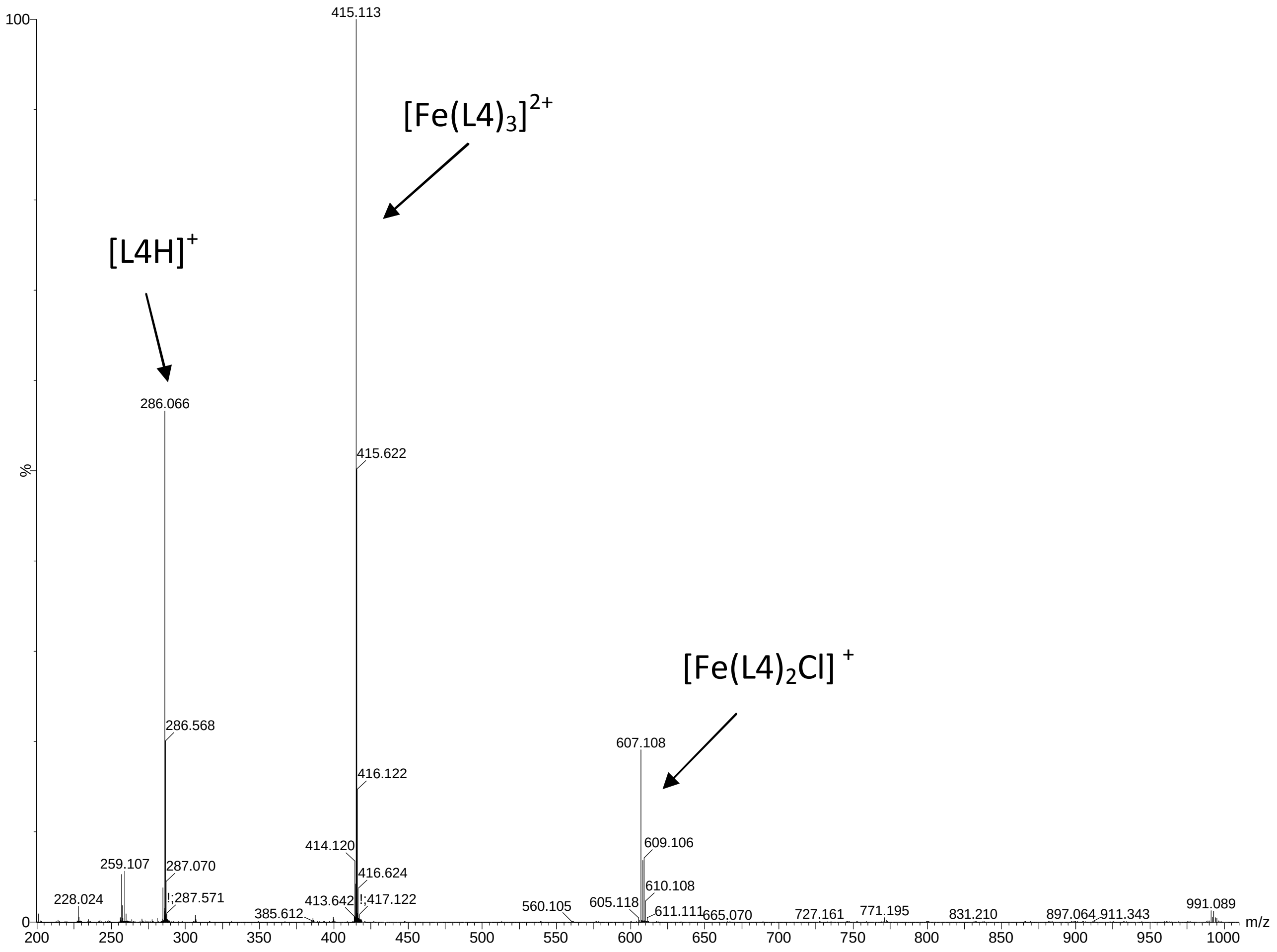

Supplementary Figure 3. ESI-MS spectrum of ligand L4 with $FeCl_2$ (acetonitrile as solvent), metal ion concentration 50 µmol L^{-1}.

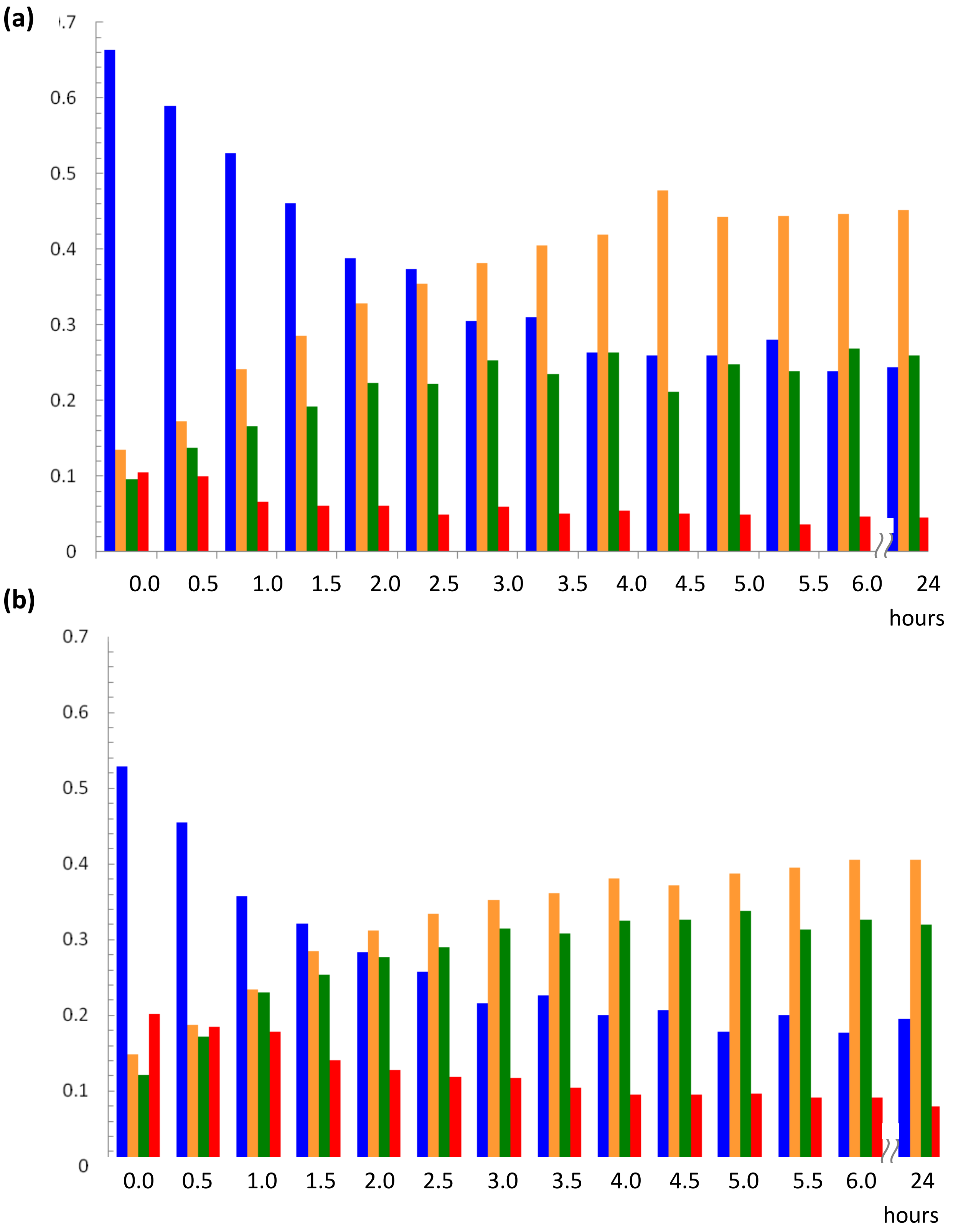

Supplementary Figure 4. Time course graphs of normalized intensity obtained for $FeBr_2$:L1:L3, determined for a) $\{[Fe(L1/L3)_3]Br\}^+$ species and b) $[Fe(L1/L3)_3]^{2+}$ $[Fe(L1)_3]$ (blue), $[Fe(L1)_2(L3)]$ (orange), $[Fe(L1)(L3)_2]$ (green) and $[Fe(L3)_3]$ (red).

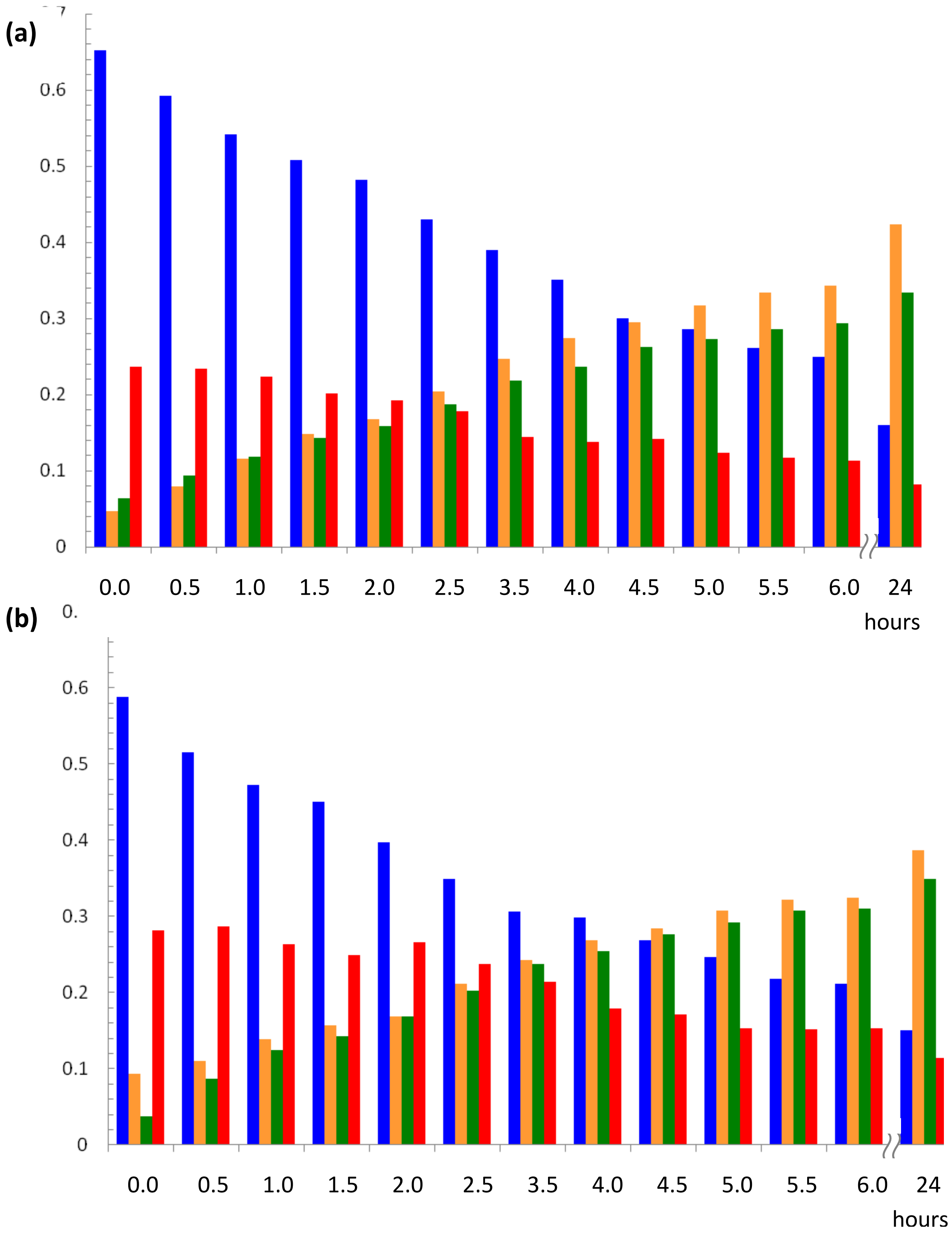

Supplementary Figure 5. Time course graphs of normalized intensity obtained for $Fe(ClO_4)_2$:L1:L3, determined for a) $\{[Fe(L1/L3)_3]ClO_4\}^+$ species and b) $[Fe(L1/L3)_3]^{2+}$ $[Fe(L1)_3]$ (blue), $[Fe(L1)_2(L3)]$ (orange), $[Fe(L1)(L3)_2]$ (green) and $[Fe(L3)_3]$ (red).

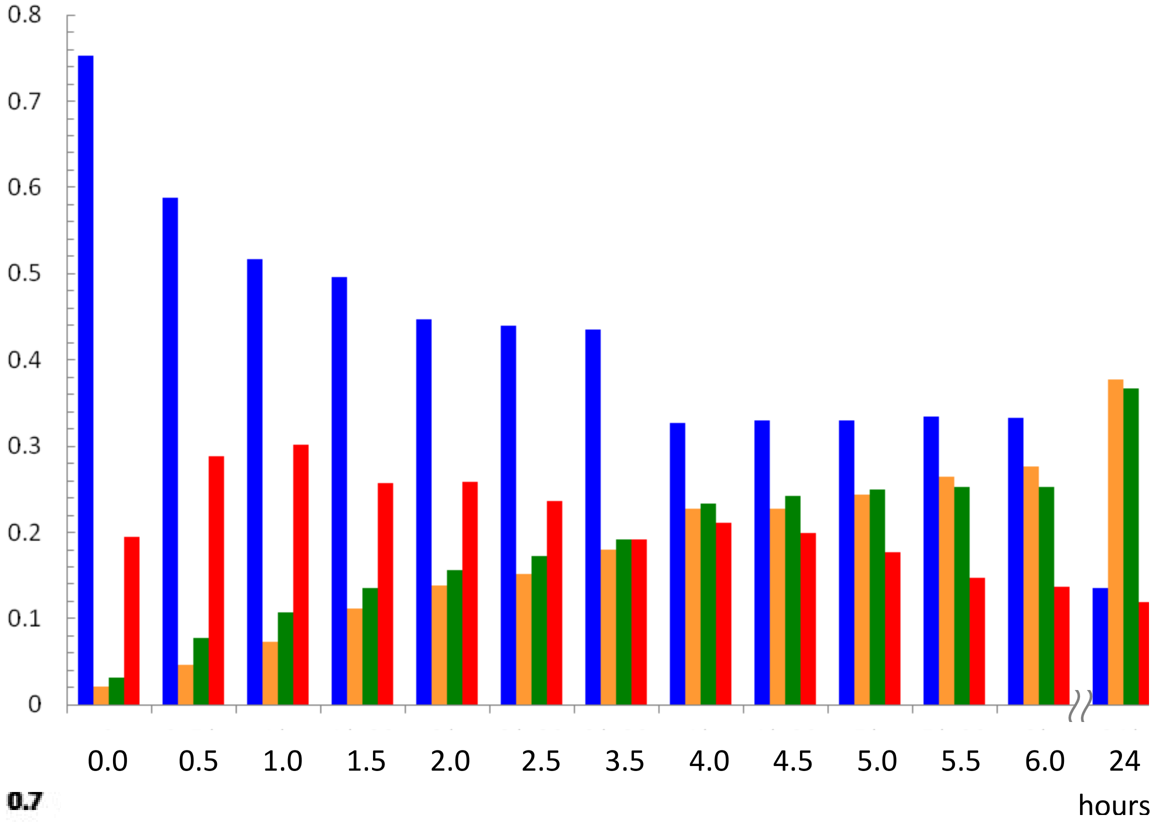

(b)

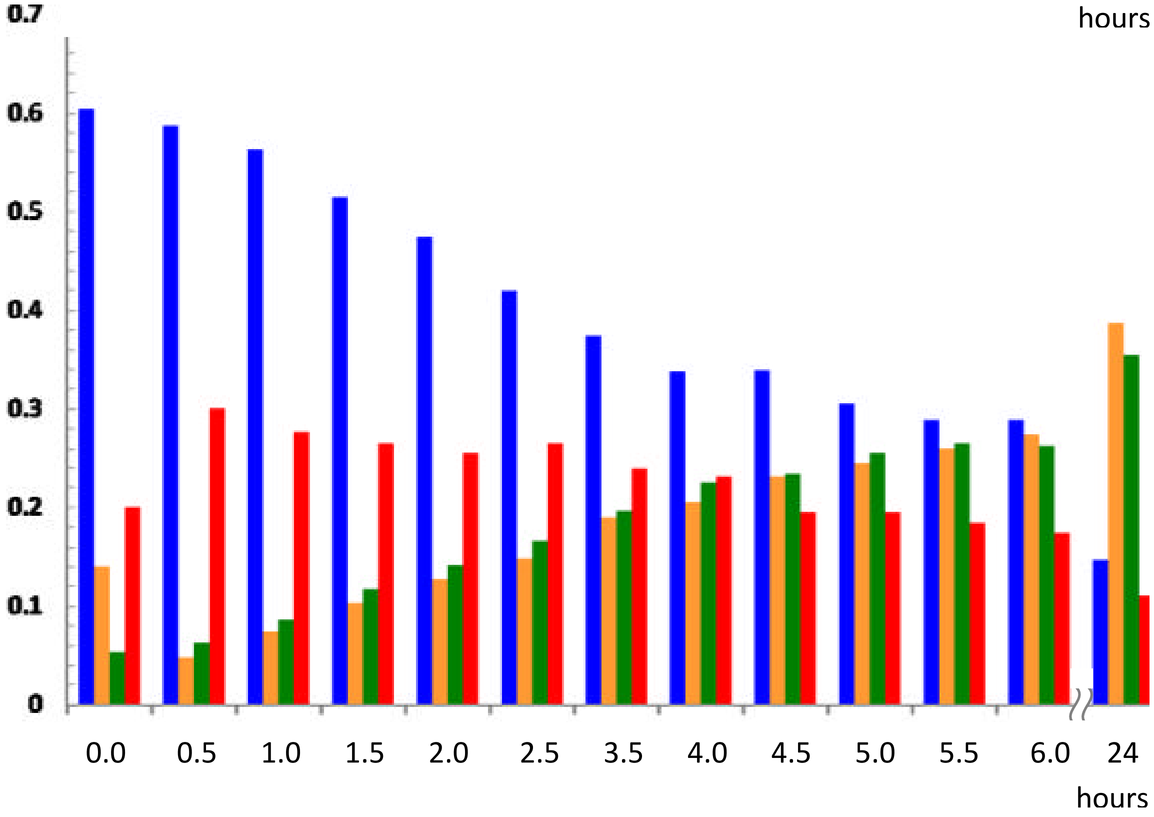

Supplementary Figure 6. Time course graphs of normalized intensity obtained for $Fe(BF_4)_2$:L1:L3, determined for a) $\{[Fe(L1/L3)_3]BF_4\}^+$ species and b) $[Fe(L1/L3)_3]^{2+}$ $[Fe(L1)_3]$ (blue), $[Fe(L1)_2(L3)]$ (orange), $[Fe(L1)(L3)_2]$ (green) and $[Fe(L3)_3]$ (red).

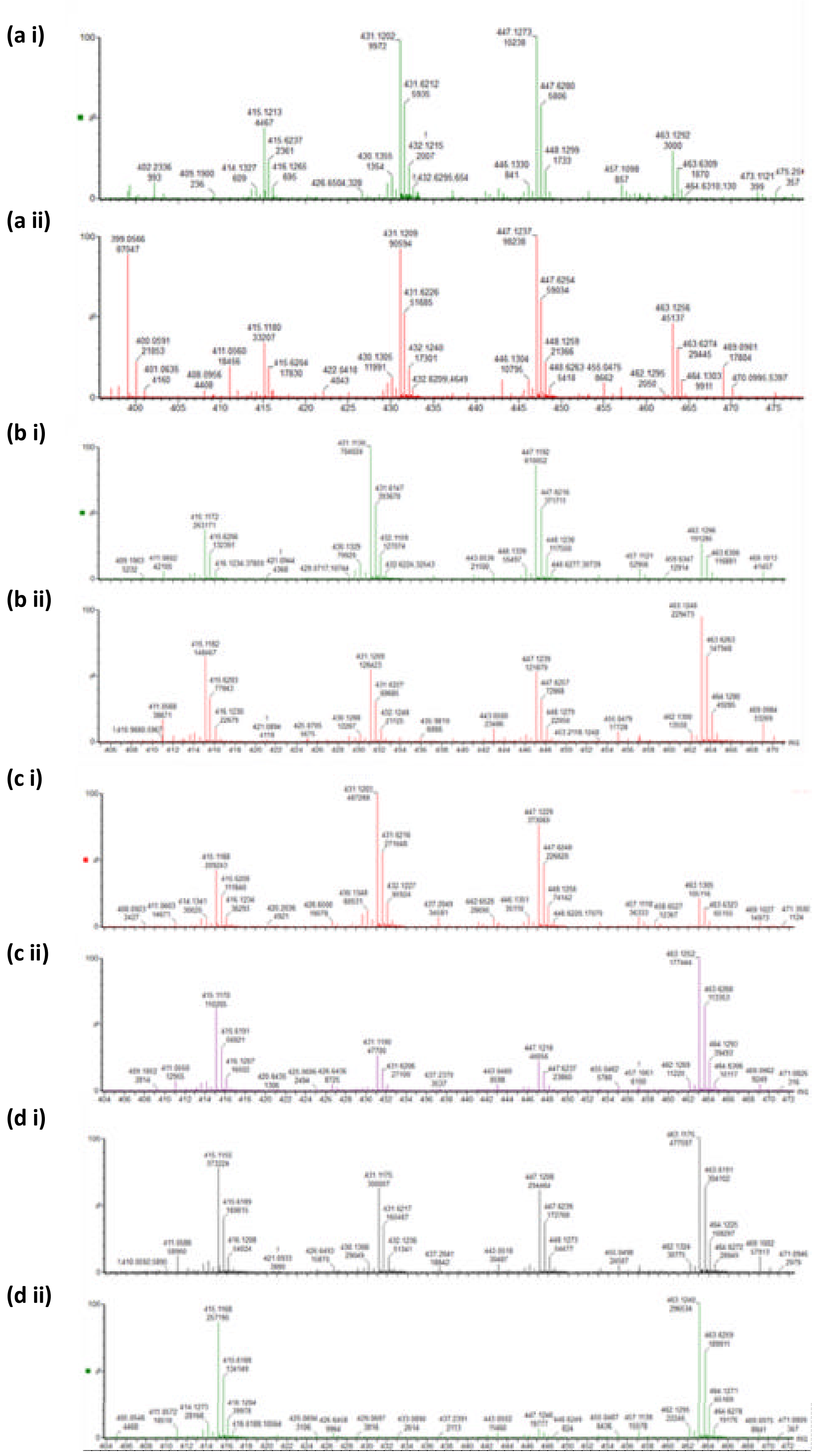

Supplementary Figure 7. ESI-MS spectra of ligands L2 and L4 with (a) $FeCl_2$, (b) $FeBr_2$, (c) $Fe(ClO_4)_2$ and (d) $Fe(BF_4)_2$ after (i) 24 hours and (ii) 5 minutes (CH_3CN as solvent), metal ion concentration 50 μmol L^{-1}.

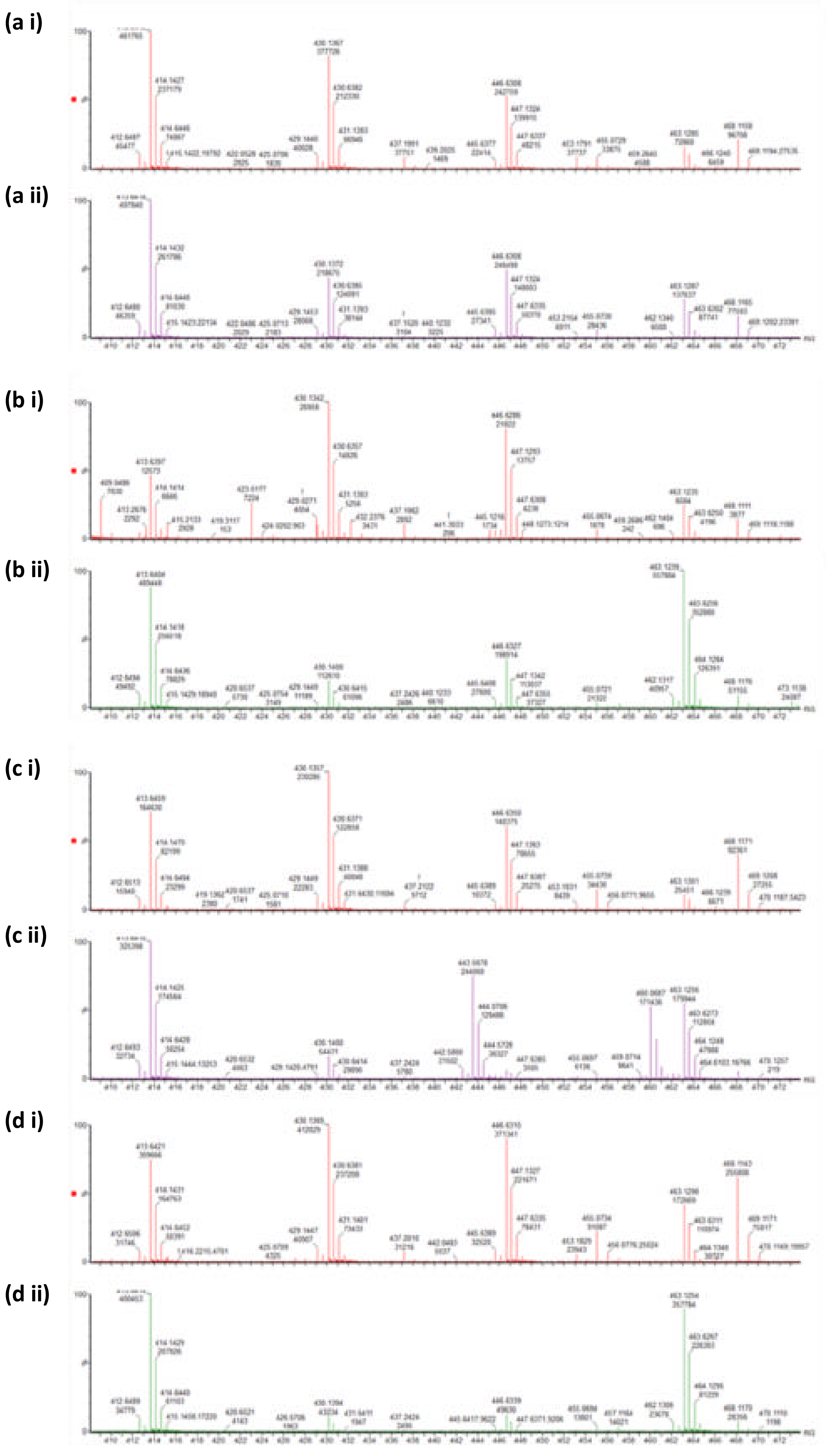

Supplementary Figure 8. ESI-MS spectra of ligands L2 and L3 with (a) $FeCl_2$, (b) $FeBr_2$, (c) $Fe(ClO_4)_2$ and (d) $Fe(BF_4)_2$ after (i) 24 hours and (ii) 5 minutes (CH_3CN as solvent), metal ion concentration 50 μmol L^{-1}.

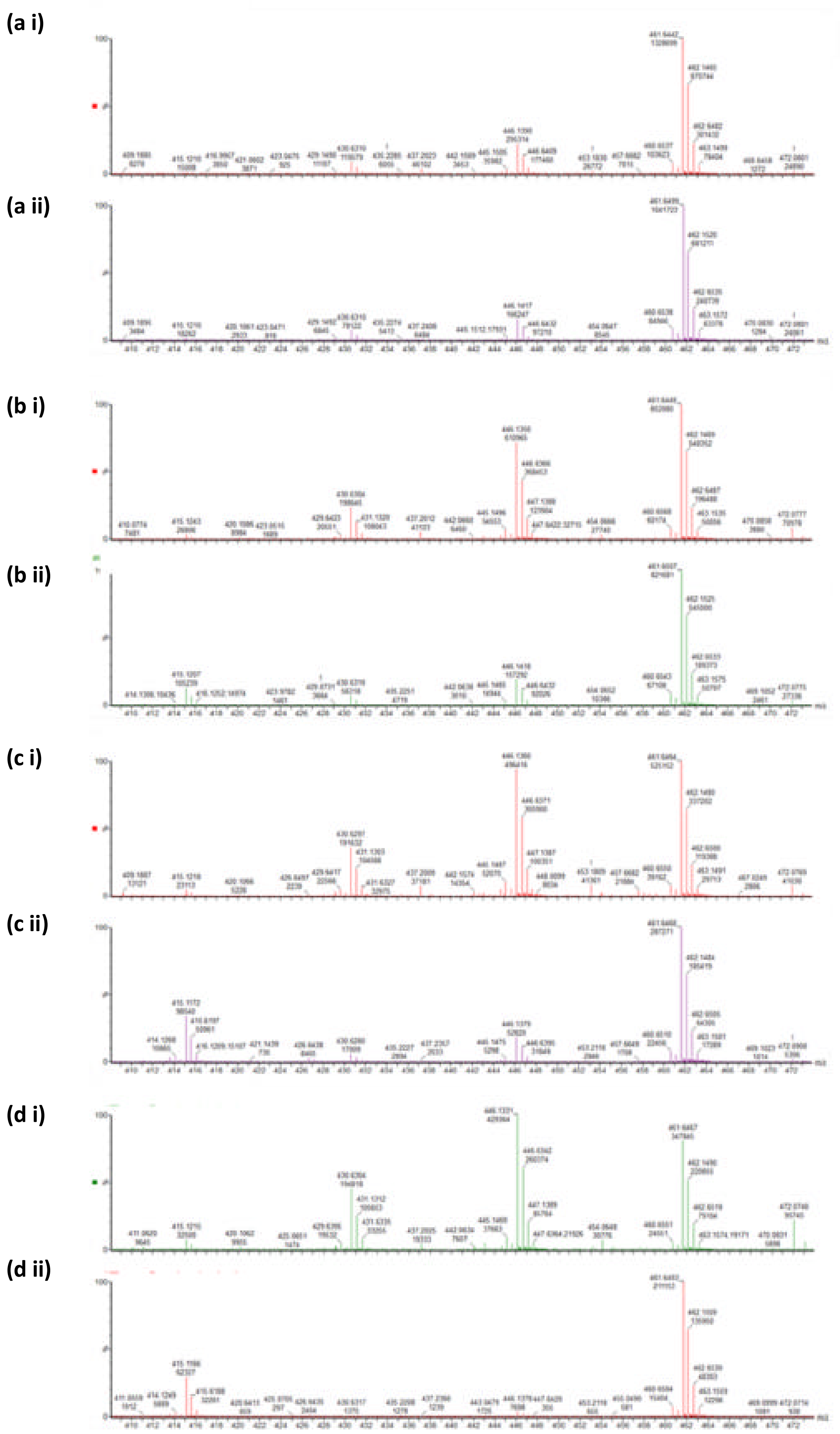

Supplementary Figure 9. ESI-MS spectra of ligands L1 and L4 with (a) $FeCl_2$, (b) $FeBr_2$, (c) $Fe(ClO_4)_2$ and (d) $Fe(BF_4)_2$ after (i) 24 hours and (ii) 5 minutes (CH_3CN as solvent), metal ion concentration 50 µmol L^{-1}.

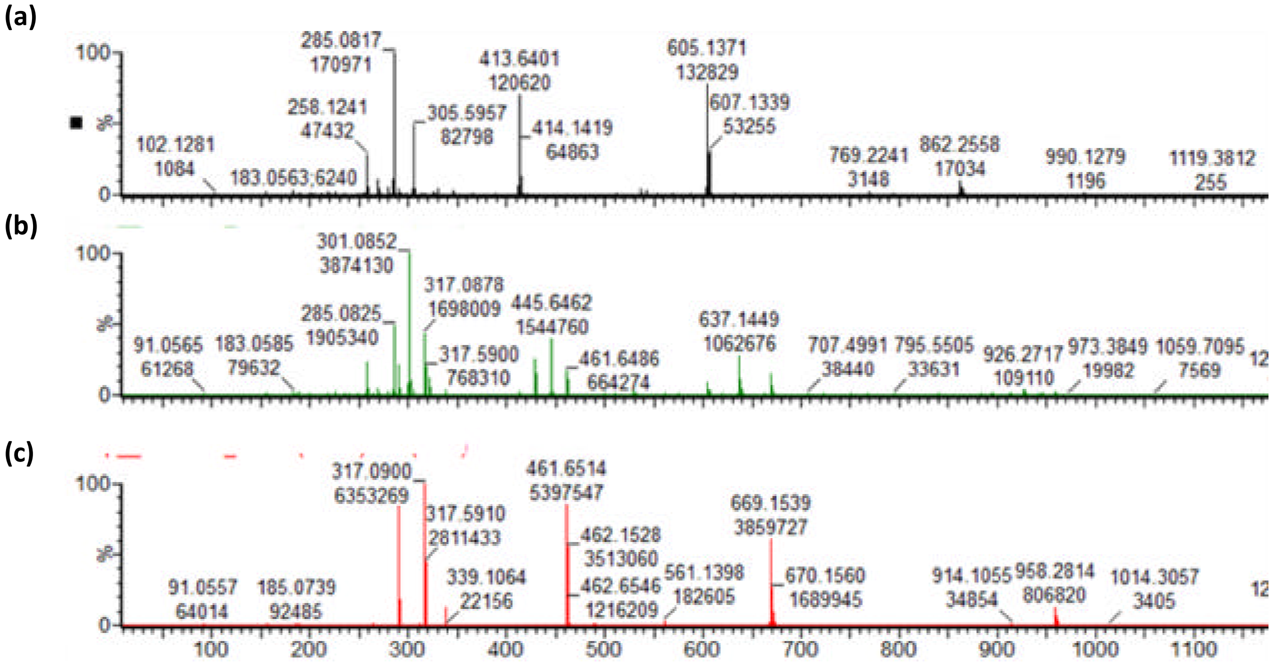

Supplementary Figure 10. ESI-MS spectra of premixed ligands L1 and L3 with $FeCl_2$ in (a) a 10:0, (b) a 5:5 and (c) a 0:10 ratio after 1 hour equilibrium (acetonitrile as solvent), metal ion concentration 50 µmol L^{-1}.

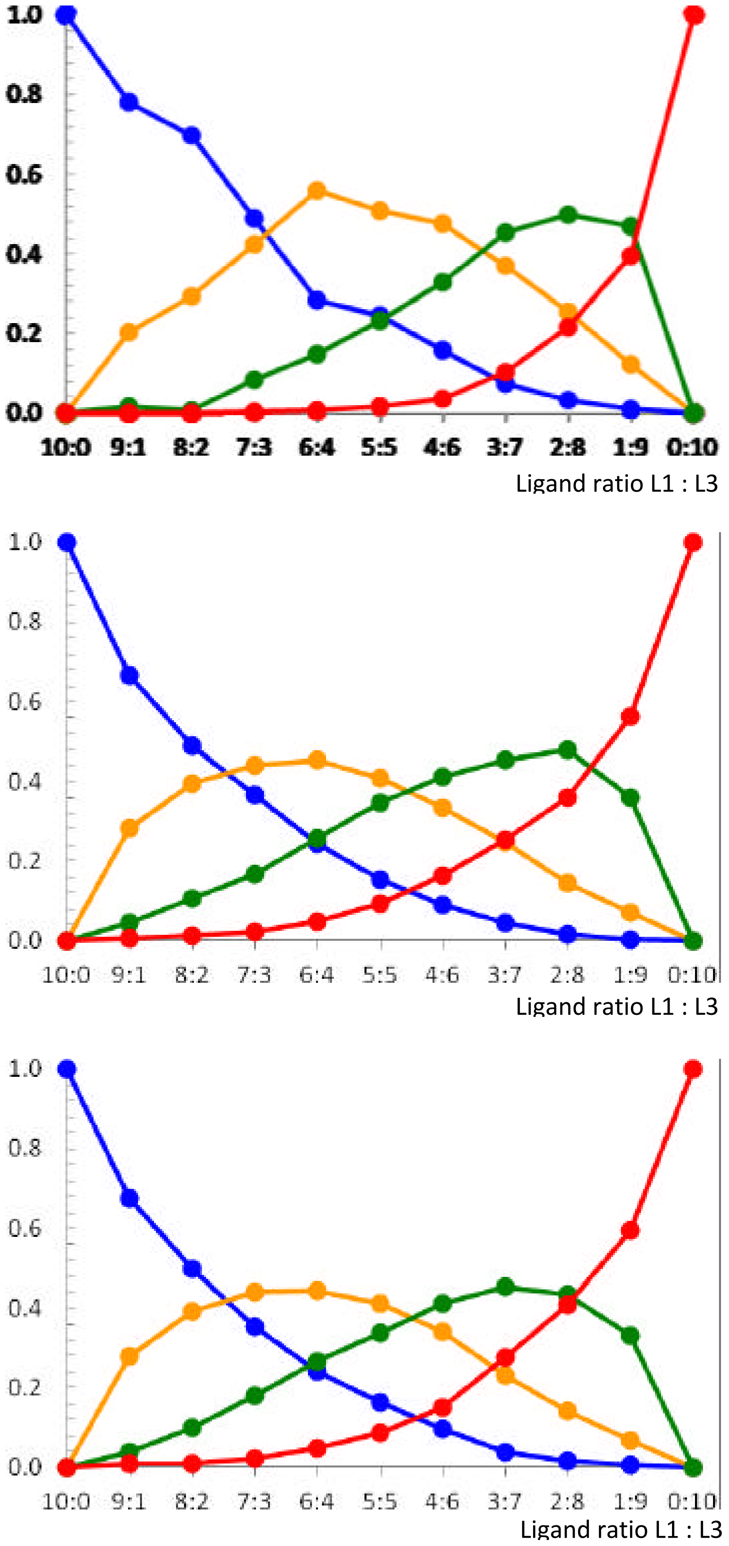

Supplementary Figure 11. Speciation plots of $\{[Fe(L1/L3)_3]X\}^+$ determined from the mass spectroscopy data with varying concentrations of ligands L1 and L3 in the presence of (a) $FeCl_2$, (b) $Fe(ClO_4)_2$ and (c) $Fe(BF_4)_2$. $[Fe(L1)_3]$ (blue), $[Fe(L1)_2(L3)]$ (orange), $[Fe(L1)(L3)_2]$ (green) and $[Fe(L3)_3]$ (red).

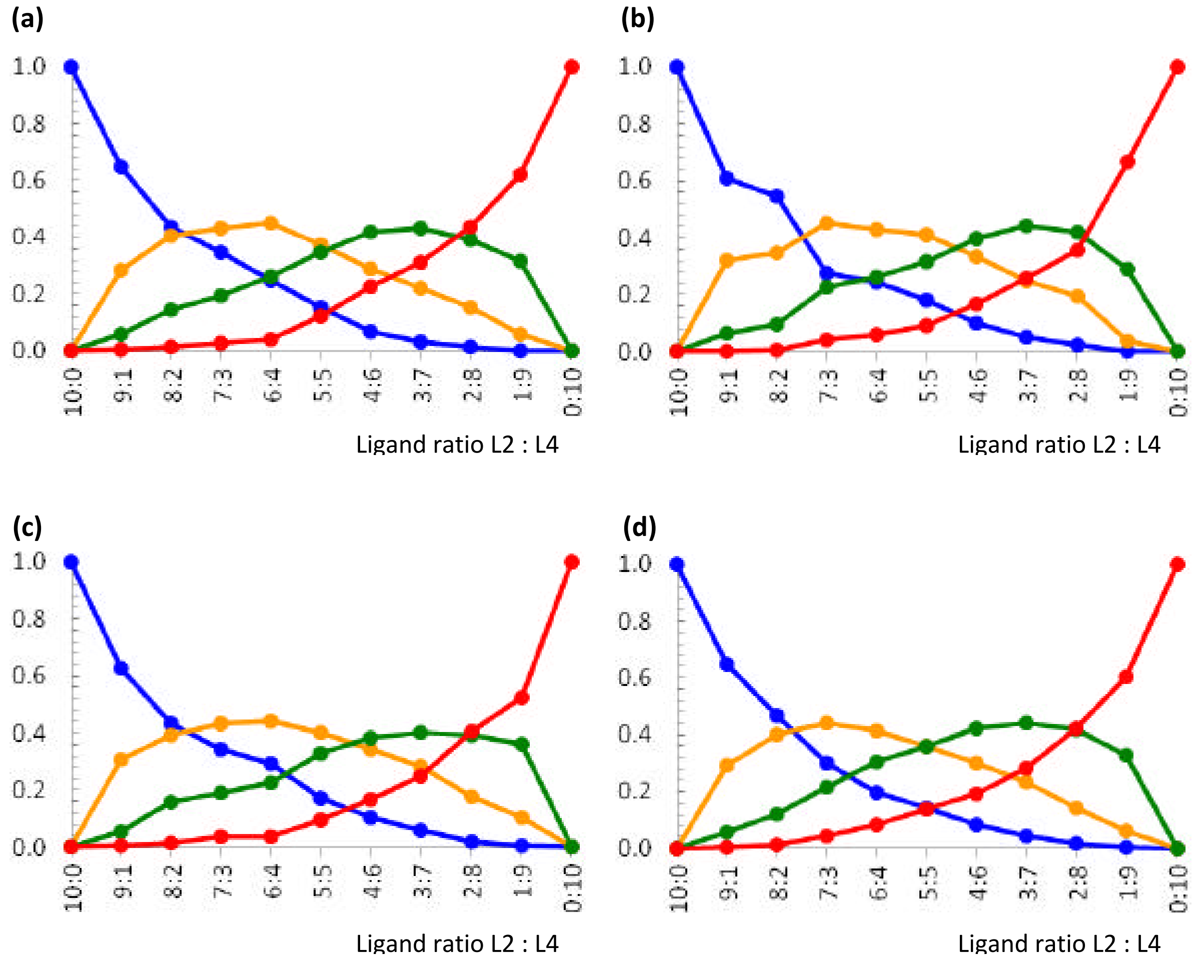

Supplementary Figure 12. Speciation plots of $[Fe(L2/L4)_3]^{2+}$ determined from the mass spectroscopy data with varying concentrations of ligands L1 and L3 in the presence of (a) $FeCl_2$, (b) $FeBr_2$, (c) $Fe(ClO_4)_2$ and (d) $Fe(BF_4)_2$. $[Fe(L2)_3]$ (blue), $[Fe(L2)_2(L4)]$ (orange), $[Fe(L2)(L4)_2]$ (green) and $[Fe(L4)_3]$ (red).

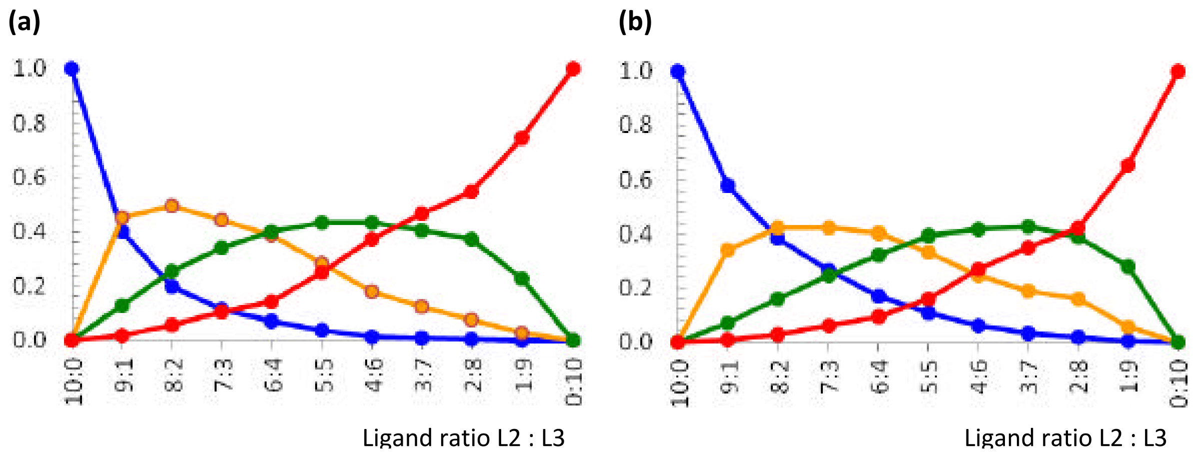

Supplementary Figure 13. Speciation plots of (a) $[Fe(L2/L3)_3]^{2+}$ and (b) $\{[Fe(L2/L3)_3]BF_4\}^+$ determined from the mass spectroscopy data with varying concentrations of ligands L2 and L3 in the presence of $Fe(BF_4)_2$. $[Fe(L2)_3]$ (blue), $[Fe(L2)_2(L3)]$ (orange), $[Fe(L2)(L3)_2]$ (green) and $[Fe(L3)_3]$ (red)**.**

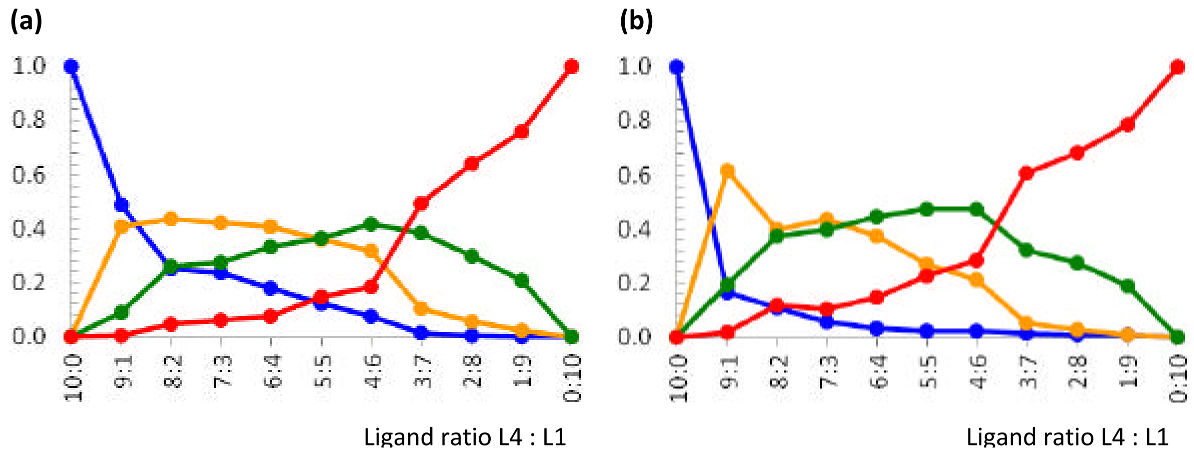

Supplementary Figure 14. Speciation plots of (a) $[Fe(L4/L1)_3]^{2+}$ and (b) $\{[Fe(L4/L1)_3]BF_4\}^+$ determined from the mass spectroscopy data with varying concentrations of ligands L4 and L1 in the presence of $Fe(BF_4)_2$. $[Fe(L4)_3]$ (blue), $[Fe(L4)_2(L1)]$ (orange), $[Fe(L4)(L1)_2]$ (green) and $[Fe(L1)_3]$ (red)**.**

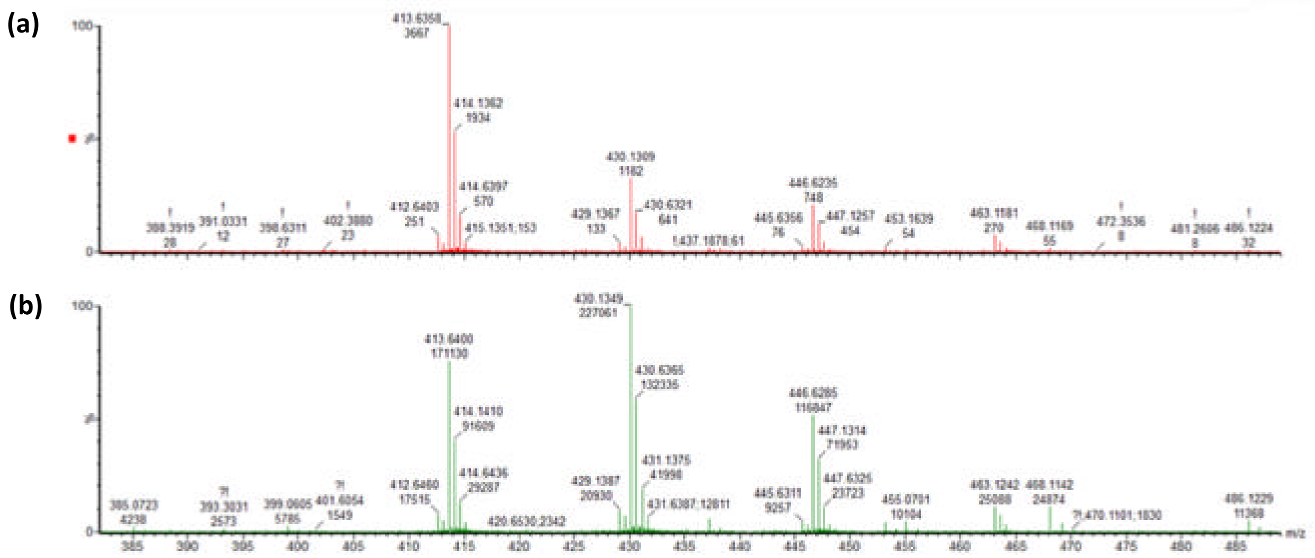

Supplementary Figure 15. ESI-MS spectra of premixed equimolar quantities of ligands L2 and L3 with $Fe(BF_4)_2$ in (a) the presence of 10 equivalents of tetrabutylammonium chloride and (b) without tetrabutylammonium chloride, after 1 hour equilibrium (acetonitrile as solvent), metal ion concentration 50 μmol L^{-1}.

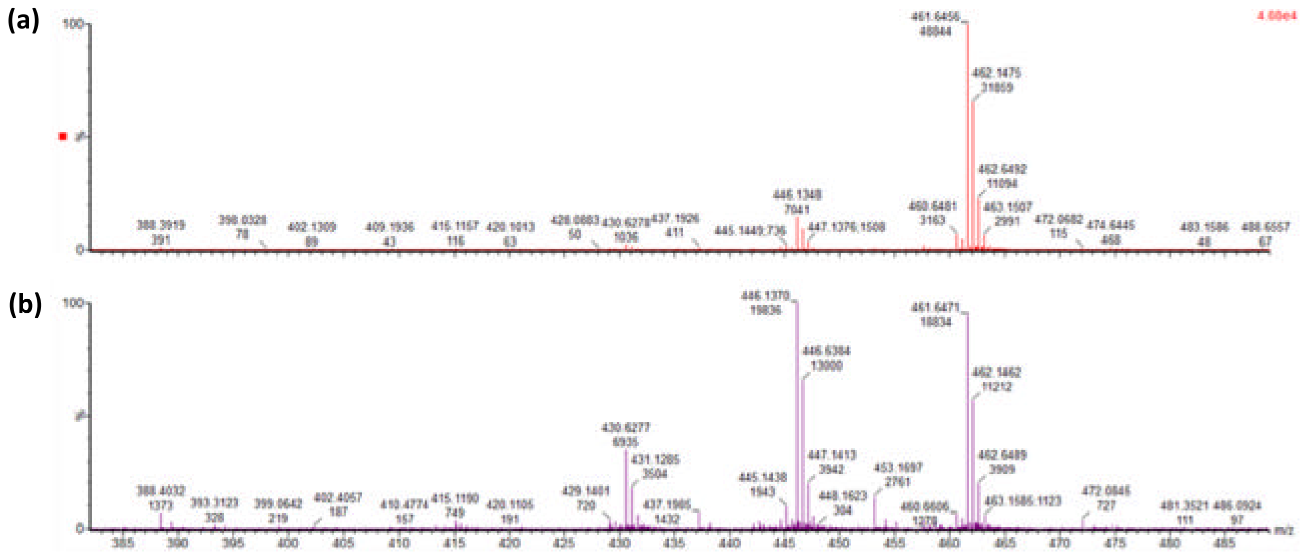

Supplementary Figure 16. ESI-MS spectra of premixed equimolar quantities of ligands L1 and L4 with $Fe(BF_4)_2$ in (a) the presence of 10 equivalents of tetrabutylammonium chloride and (b) without tetrabutylammonium chloride, after 1 hour equilibrium (acetonitrile as solvent), metal ion concentration 50 μmol L^{-1}.

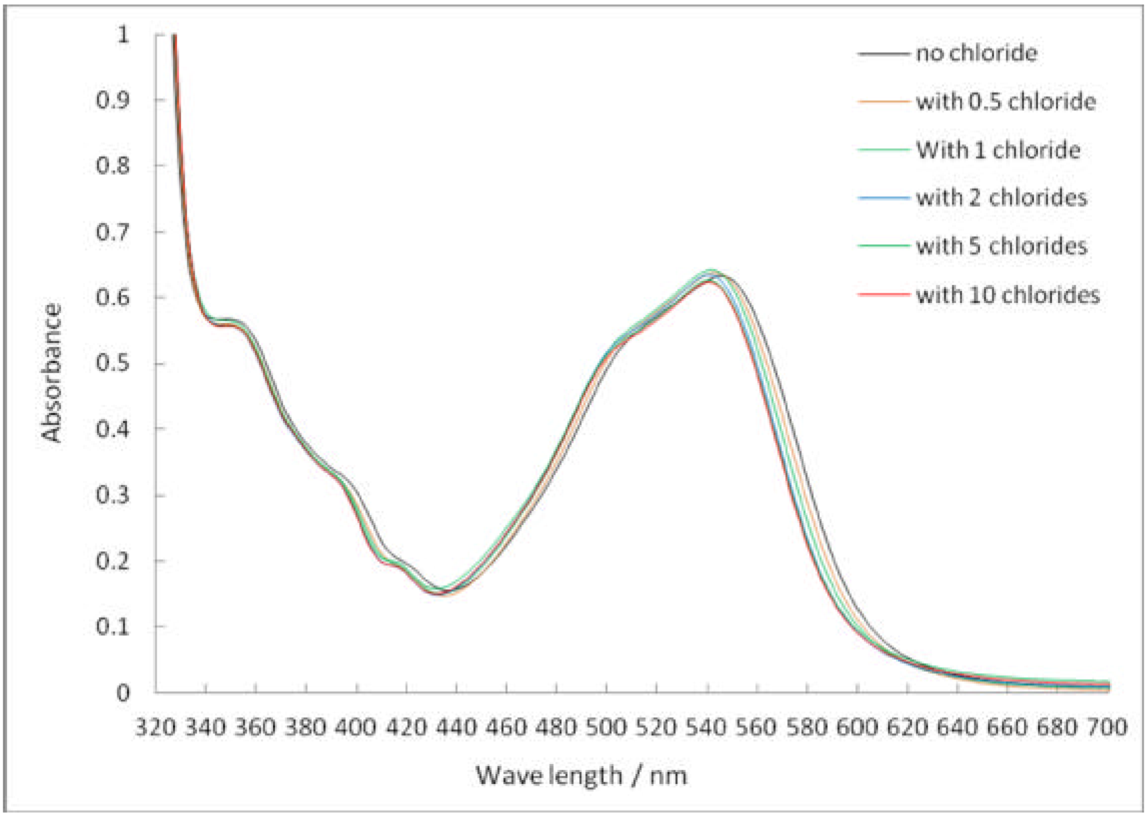

Supplementary Figure 17. UV / vis spectrum spectra of premixed equimolar quantities of ligands L1 and L4 (5×10^{-4} mol L^{-1}) with $Fe(BF_4)_2$ (1×10^{-4} mol L^{-1}) in (a) the presence of increasing equivalents of tetrabutylammonium chloride (0 to 1×10^{-3} mol L^{-1}) and after 1 hour equilibrium (acetonitrile as solvent).
